# Anatomical and Surgical Landmarks for Facial Nerve Identification During Parotidectomy: A Critical Narrative Review of Reliability, Clinical Utility, and Operative Decision-Making

**DOI:** 10.3390/jcm15218718

**Published:** 2026-11-08

**Authors:** Nina Szczerba, Łukasz Olewnik, Piotr Łabętowicz, Jolanta Łuniewska-Bury, Magdalena Ciechanowska, Daniel Casanova-Martínez, Marta Pośnik, Ingrid C. Landfald

**Affiliations:** 1 Department of Clinical Anatomy, Mazovian University in Płock, 09-402 Płock, Poland; n.szczerba@mazowiecka.edu.pl (N.S.); l.olewnik@mazowiecka.edu.pl (Ł.O.); 2 “VARIA” Research Laboratory, Mazovian University in Płock, 09-402 Płock, Poland; 3 “VARIANTIS” Research Laboratory, Mazovian University in Płock, 09-402 Płock, Poland; p.labetowicz@mazowiecka.edu.pl (P.Ł.);; 4 Department of Forensic Medicine, Pathology and Histology, Mazovian University in Płock, 09-402 Płock, Poland; 5 Faculty of Medicine, Collegium Medicum, Mazovian University in Płock, 09-402 Płock, Poland; j.luniewska-bury@wyk.mazowiecka.edu.pl; 6 Faculty of Health Sciences, Collegium Medicum, Mazovian University in Płock, 09-402 Płock, Poland; m.ciechanowska@mazowiecka.edu.pl; 7 Facultad de Medicina, Universidad de Valparaíso, Campus San Felipe, San Felipe 2170000, Chile

**Keywords:** tympanomastoid suture, tragal pointer, posterior belly of digastric muscle, extracranial neuroanatomy, anatomical variation, retrograde dissection, nerve preservation, surgical navigation

## Abstract

**Background:** Facial nerve identification during parotidectomy requires interpretation of complementary landmarks. This critical narrative review distinguishes anatomical proximity from operative accessibility and evidence of improved facial nerve preservation. **Methods:** PubMed/MEDLINE, Scopus and Web of Science were searched through 7 October 2026, with a further search during revision and records reconciled on 2 November 2026. Two authors screened the literature and evaluated study methods and clinical applicability. Screening of 7157 deduplicated November records added no studies. The final bibliography contains 80 distinct publications; 22 principal morphometric reports are tabulated with cadaveric and intraoperative measurements separated. **Results:** Reported landmark distances depend on reference points, neural endpoints and study setting. Mean tympanomastoid suture–trunk distances differed from 2.5 mm in one cadaveric series to 10.0 mm in another. In 32 operations for benign superficial-lobe masses, the tragal pointer, posterior belly of digastric and suture were exposed in 32, 26 and 25 cases, respectively. Comparative evidence on dissection direction predominantly concerns benign disease, limiting extrapolation to revision, malignant and irradiated fields. **Conclusions:** Complementary landmarks support anatomical orientation, but proximity and concordance do not establish a safe dissection plane or improved postoperative function. The proposed operative framework integrates landmark reassessment, alternative identification routes and adjunctive stimulation; it remains unvalidated and requires prospective evaluation against operative and functional outcomes.

## 1. Introduction

Facial nerve preservation during parotidectomy requires recognition of an extracranial trunk that divides into a variable intraparotid plexus [1,2,3]. The surgeon may approach the trunk proximally or trace an identifiable peripheral branch back toward it.

Postoperative facial nerve dysfunction remains one of the most clinically important complications of parotidectomy. Immediate postoperative weakness must be distinguished from persistent dysfunction assessed at a specified follow-up interval. In a prospective cohort of 132 patients undergoing surgery for benign parotid neoplasms, reported facial palsy rates decreased from 40.2% on the first postoperative day to 3.9% at six months and 1.6% at twelve months [4]. In a separate series of 131 primary conservative parotidectomies for benign or malignant tumours, Gaillard et al. [5] reported dysfunction in 42.7% on the first postoperative day and none at six months. Early dysfunction was more frequent after total than superficial parotidectomy. These findings illustrate the importance of assessment timing and operative case mix: rates should be interpreted alongside baseline function, tumour characteristics, extent of resection and previous treatment, rather than treated as interchangeable estimates of permanent injury.

Early division can shorten the segment of undivided nerve available for proximal identification [1,2,3,6]. A tumour can displace this segment even when the adjacent bony landmarks remain palpable. After previous treatment, the additional difficulty may be finding a tissue plane between a recognisable landmark and the nerve.

The tympanomastoid suture lies close to the trunk in several anatomical and operative series [7,8,9,10,11]. For the surgeon, an equally important question is whether the suture can be recognised before the trunk is exposed. Studies that measure distance only after finding both structures cannot answer that question.

Ji et al. synthesised published distances from landmarks to the trunk [12]. Łabętowicz et al. reviewed facial nerve anatomy and preservation across surgical settings [13]. We focus on parotidectomy and ask which points were measured, how frequently each landmark could be identified during exposure, and whether using it was associated with better facial function. We apply these questions separately to primary surgery and previously treated fields.

## 2. Materials and Methods

### 2.1. Literature Search Strategy

PubMed/MEDLINE, Scopus and Web of Science were searched from database inception through 7 October 2026. Non-English reports were eligible when their full texts could be assessed in translation.

During revision, a further search of all three databases was undertaken, with the exported records reconciled on 2 November 2026. This yielded 13,541 records: 3991 from PubMed, 6538 from Scopus and 3012 from Web of Science. After deduplication, 7157 records remained. Two authors screened titles and abstracts, assessed relevant full texts and jointly determined eligibility. No additional studies were incorporated from this screening.

The search workbook documents retrieval and deduplication but does not retain individual eligibility decisions or full-text assessment and exclusion counts. The documented search strategies and selection summary are provided in Supplementary Methods S1.

### 2.2. Study Selection and Evidence Evaluation

Two authors screened titles, abstracts and relevant full texts, analysed the evidence and jointly decided inclusion or exclusion. They extracted information together and resolved differences by discussion. Eligible primary reports described a defined landmark, neural endpoint, identification technique, performance against operative findings, or facial function after parotid surgery. Comparative studies, surgical series and anatomically informative variants were interpreted according to design. Reviews supplied context and citation leads. Isolated intracranial or intratemporal observations without relevance to extracranial identification were excluded from morphometric comparison. Experimental optical imaging was considered separately from the human evidence.

The corrected bibliography contains 80 distinct publications, including contextual reviews and other supporting sources; this is not a count of primary studies selected through a reconstructable systematic-review flow. Table S1 presents 22 principal primary morphometric reports in 26 rows, with separate cadaveric and clinical entries for four publications. Duplicate bibliographic entries were consolidated by source identity, and potentially overlapping cohorts were flagged separately. Historical screening and exclusion totals could not be recovered.

Evidence was evaluated according to landmark definition, anatomical relationship to the facial nerve, measurement methodology, reported variability, intraoperative accessibility, and clinical applicability. Qualitative methodological appraisal considered sample selection, the distinction between donors and specimens, specimen preparation, measurement endpoints and direction, measurement reproducibility, and the identification of dispersion measures. Bilateral specimens were distinguished from independent donors.

For clinical studies, appraisal additionally considered case mix, primary versus revision surgery, extent of resection, baseline facial nerve function, outcome definitions, assessment timing, and adjustment for potential confounding. Imaging findings were interpreted according to their reference standard and the distinction between localisation accuracy, procedural agreement and postoperative nerve outcomes. Unverified details and inconsistencies within source reports are identified in Supplementary Tables S1 and S2 rather than resolved by assumption. Table S1 records the source-access basis for verification during revision and distinguishes unavailable full texts from details not reported or not clearly defined in accessible source text.

### 2.3. Comparative Synthesis

Evidence was synthesised qualitatively because of substantial heterogeneity in study designs, anatomical definitions, measurement techniques, and reported outcomes. Quantitative pooling was not undertaken, and published measurements were interpreted within the methodological context of the original investigations.

Landmark performance was assessed across three distinct dimensions: anatomical proximity, describing spatial relationships to the facial nerve; anatomical reproducibility, reflecting consistency across specimens and patients; and operative utility, encompassing landmark accessibility, recognisability, and practical value during dissection.

Reported measurements and clinical findings were distinguished from the authors’ anatomical interpretation. The accessibility and structural-behaviour descriptions in the functional comparison represent a qualitative synthesis rather than validated scores; supporting evidence types and limitations are identified in the table footnotes. Stability of a landmark itself was not assumed to establish stability of its relationship to a displaced nerve.

The proposed operative framework combines the reported evidence with context-dependent anatomical reasoning; its validation requirements are set out in Section 8 and Section 10.

## 3. Surgical Anatomy Relevant to Facial Nerve Identification

### 3.1. Extratemporal Course of the Facial Nerve

Following its emergence through the stylomastoid foramen, the facial nerve enters the extracranial compartment, where it typically gives rise to the posterior auricular nerve and motor branches to the posterior belly of the digastric and stylohyoid muscles before entering the posteromedial aspect of the parotid gland [13,14,15]. These proximal branches are surgically relevant because they arise before the major intraparotid division and may therefore serve as supplementary neural landmarks for tracing the facial nerve toward its main extracranial trunk [16].

**Figure 1:**
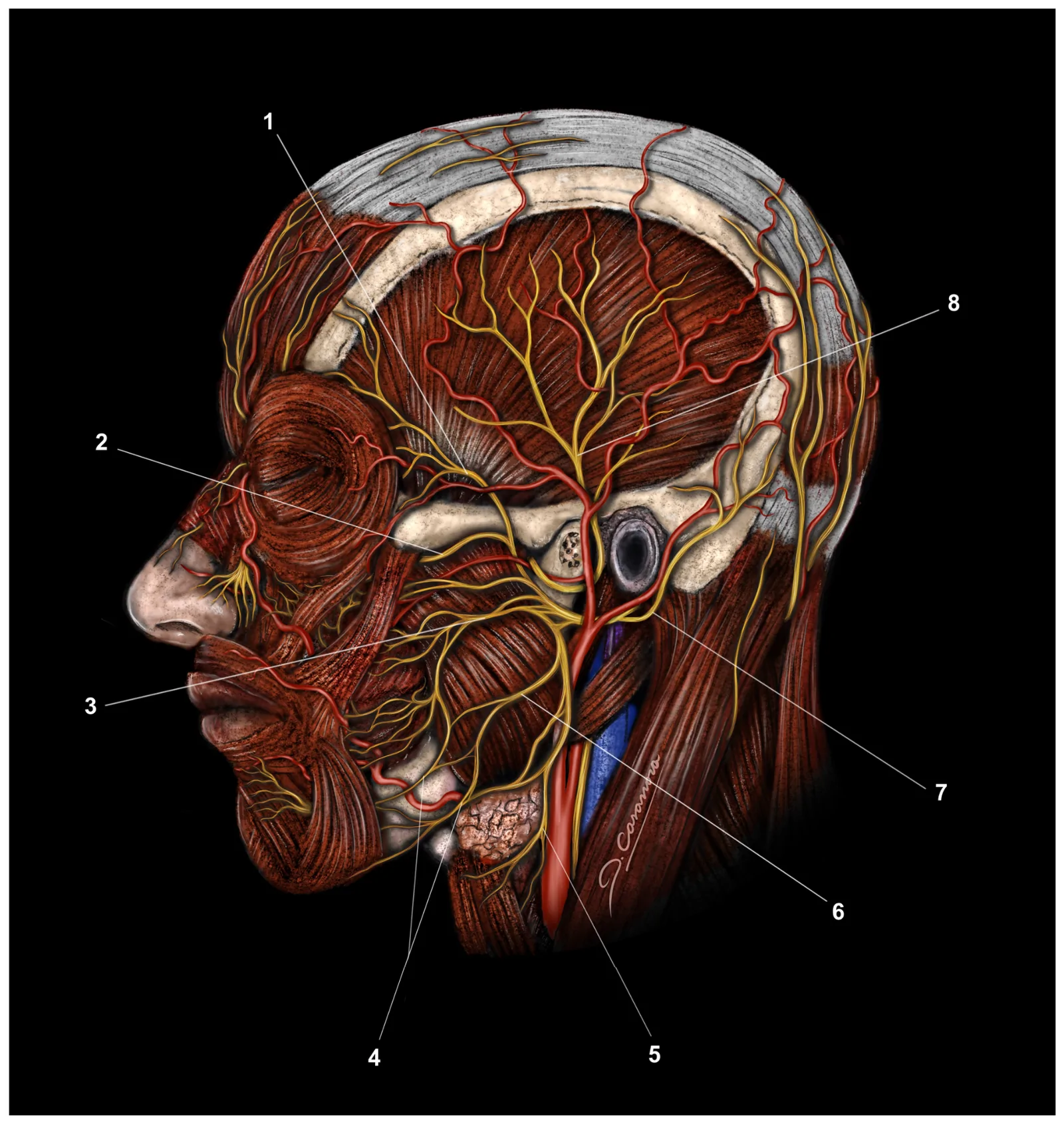
Extracranial branching pattern of the facial nerve and its anatomical relationships. Lateral view showing the temporal (1), zygomatic (2), buccal (3), marginal mandibular (4), and cervical (5) branches of CN VII; an additional facial nerve ramus crossing the lower masseter (6); the posterior auricular nerve (7); and a nerve provisionally identified as the auriculotemporal nerve (CN V3) (8).

Within the parotid gland, the facial nerve follows a short course before typically dividing into temporofacial and cervicofacial divisions, which subsequently contribute to the temporal, zygomatic, buccal, marginal mandibular, and cervical branches [1,2,3,13] (Figure 1). Considerable anatomical variability exists in trunk length, level and pattern of primary division, branching configuration, and interbranch communications, frequently resulting in formation of a complex intraparotid facial plexus [17,18,19]. Distal branching patterns primarily inform intraparotid dissection and branch preservation; they do not provide a fixed coordinate for proximal trunk identification.

Distal branch preservation also requires regional anatomical knowledge. Variation in zygomaticus muscle morphology concerns the distal motor apparatus [20,21,22], while temporal fascial relationships inform protection of the temporal (frontal) branch during temporalis flap elevation [23]. Temporalis morphology is relevant to flap planning [24,25]. These considerations do not define the location of the proximal trunk.

Proximal identification instead depends on the nerve’s emergence from the stylomastoid foramen and its relationships with adjacent osseous, muscular, cartilaginous, vascular and neural structures.

### 3.2. Surgical Exposure of the Facial Nerve Trunk

Incision selection considers tumour size and location, anticipated pathology and the need for cervical extension. Modifications of the Blair incision aim to reduce visible scarring while retaining adequate operative exposure [26].

**Figure 2:**
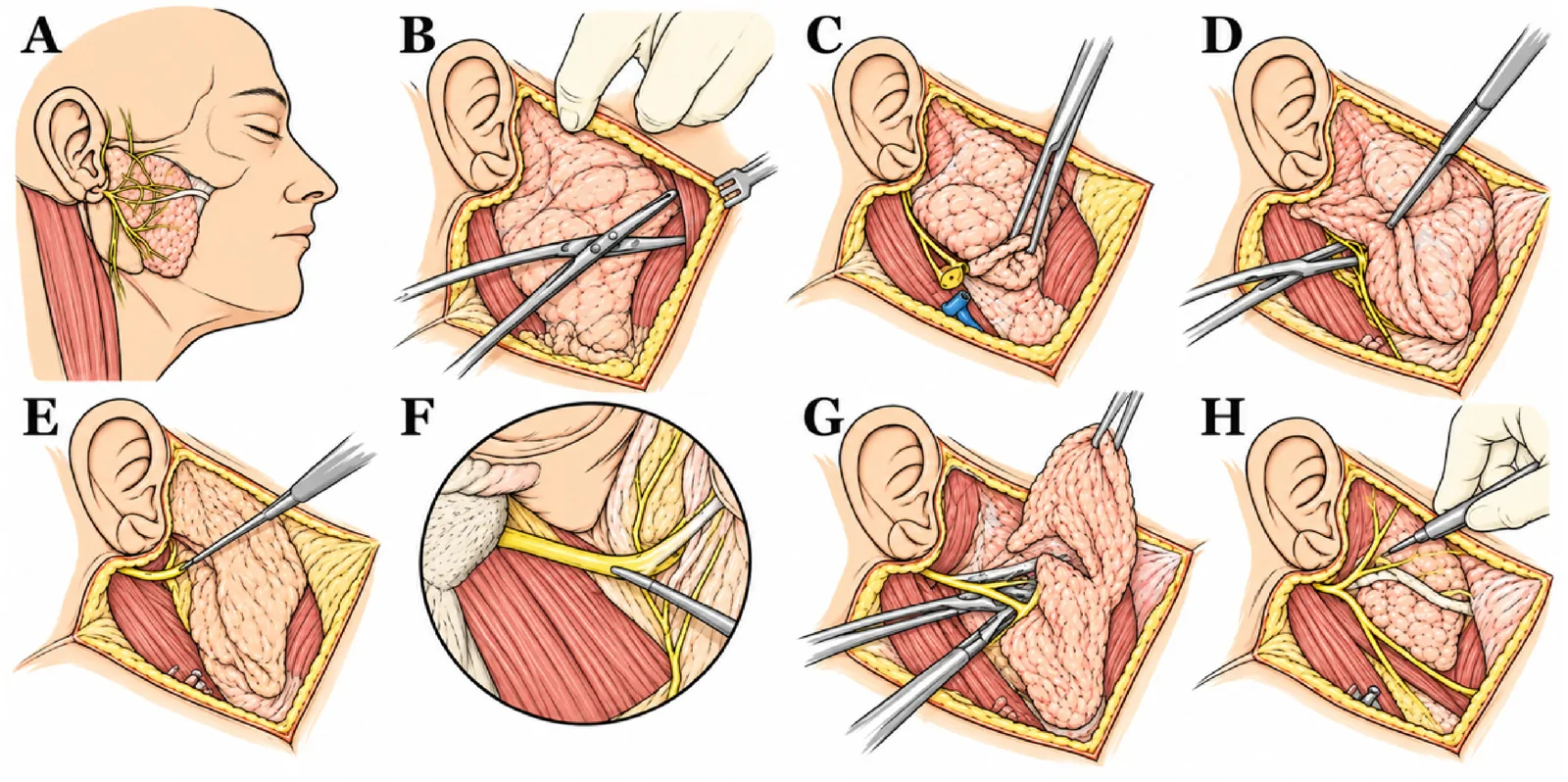
Schematic illustration of selected surgical stages of facial nerve identification and preservation during parotidectomy. (**A**) Lateral view illustrating the anatomical relationship between the facial nerve and parotid gland. (**B**) Exposure of the parotid gland following skin-flap elevation. (**C**) Mobilisation of the parotid tail, showing the divided ends of the great auricular nerve and external jugular vein in the illustrated operative sequence. (**D**) Blunt dissection to facilitate facial nerve identification. (**E**) Identification of the facial nerve trunk using a nerve-stimulation probe. (**F**) Magnified view of the facial nerve trunk and adjacent anatomical landmarks. (**G**) Mobilisation of parotid tissue with blunt dissection around the exposed facial nerve branches. (**H**) Stimulation of exposed facial nerve branches following tumour removal. Nerves are depicted in yellow. Illustrations are schematic and not to scale.

Following elevation of the skin flap and exposure of the parotid region (Figure 2), structures including the sternocleidomastoid muscle, great auricular nerve, mastoid region, posterior belly of the digastric muscle, and tragal cartilage provide progressive anatomical orientation toward the facial nerve trunk. The sequence in which these structures are encountered varies according to the incision, extent of exposure, tumour location, and preferred operative technique. Anterograde dissection identifies the main trunk before following its divisions distally; retrograde dissection identifies a peripheral branch and follows it proximally towards the trunk.

The published C–M–S technique identifies cartilage, muscle and suture sequentially before locating the trunk [27]. Conventional proximal dissection uses the sternocleidomastoid muscle and posterior belly of the digastric muscle for inferior orientation towards the stylomastoid region [28].

Comparative evidence does not establish universal superiority of either approach across all operative settings. Stankovic et al. [29] synthesised eight publications and found no statistically significant difference in temporary or permanent facial nerve palsy: the anterograde-versus-retrograde odds ratios were 2.64 (95% confidence interval [CI], 0.97–7.21) and 4.31 (95% CI, 0.44–42.28), respectively. Mashrah et al. [30] included 570 patients across ten reports, comprising 319 retrograde and 251 anterograde procedures; studies involving malignant tumours, revision surgery or recurrent disease were excluded. Among 481 patients contributing facial nerve outcomes, the anterograde-versus-retrograde risk ratios were 1.16 (95% CI, 0.80–1.67) for transient dysfunction and 4.63 (95% CI, 0.72–29.04) for permanent dysfunction. These nonsignificant findings, particularly the wide intervals for permanent dysfunction, do not demonstrate equivalence.

More recent evidence has suggested a potential advantage of retrograde dissection in selected benign parotid surgery. Smith et al. [31] reported temporary facial nerve paresis in 3/35 retrograde cases (8.6%) compared with 30/70 anterograde cases (42.9%; *p* < 0.001) undergoing superficial parotidectomy for benign tumours. Although groups were balanced for tumour size and location, the retrospective design and differing histological composition limit causal interpretation. Kim et al. [32] included 21 studies and 1632 patients and reported lower odds of transient palsy with retrograde dissection (odds ratio, 0.73; 95% CI, 0.54–0.98), principally in the superficial-parotidectomy subgroup, without a statistically significant difference in permanent palsy.

These comparisons concern dissection direction rather than the independent performance of individual landmarks. They do not establish greater safety specifically in scarred revision, malignant or irradiated fields. Selection of the approach should therefore remain conditional on tumour location, surgical extent, preserved anatomy and surgeon experience. Study populations, effect estimates and limitations are summarised in Supplementary Table S2.

The following sections examine the anatomical relationships underlying these approaches, with study-specific measurements and limitations presented in Table S1 [10,27,33,34].

## 4. Principles of Surgical Landmark Selection

Successful identification of the facial nerve trunk during parotid surgery depends not only on detailed anatomical knowledge but also on selection and interpretation of reliable intraoperative reference structures. In this review, landmark performance is considered across four domains: anatomical constancy; surgical accessibility and proximity; resistance to anatomical distortion; and reproducibility with clinical applicability. These domains provide an interpretative framework rather than a validated scoring system [14,15,33]. Measured properties are distinguished from descriptive surgical observations and the authors’ anatomical interpretation.

### 4.1. Anatomical Constancy

Constancy describes how often a specified structure or configuration is present in the examined material. For each estimate, the denominator matters. A study that excludes an unidentifiable landmark can describe distances among successful exposures but cannot establish how often the landmark is available in an unselected operation.

Conversely, anatomically variable structures may remain surgically useful when their relationship with the facial nerve is interpreted together with additional reference points. Anatomical constancy should therefore be considered as one component of landmark performance rather than as an isolated criterion for determining clinical utility [16,19,33].

### 4.2. Surgical Accessibility and Proximity to the Facial Nerve

Accessibility describes when a landmark can be seen or palpated and what dissection is needed to reach it. Operative reports can quantify accessibility by recording successful identification among all attempted exposures. Proximity is a different measurement, taken between a defined point on the landmark and a defined neural endpoint.

In a combined cadaveric and clinical investigation, Saha et al. identified the posterior belly of the digastric muscle as the most readily recognisable landmark during live surgery, while the tympanomastoid suture lay closer to the facial nerve trunk. These observations illustrate the distinction between accessibility and proximity; they do not establish that either property independently improves postoperative nerve function [10].

Proximity alone should therefore not be interpreted as synonymous with reliability. Where accessibility has not been assessed using identification time, recognition rates or another explicit outcome, descriptions of ease of exposure remain qualitative surgical observations or anatomical judgments [27,33,34].

### 4.3. Resistance to Anatomical Distortion

Resistance to distortion comprises two separate properties: preservation of the landmark’s recognisability and preservation of its relationship with the facial nerve. A structure may remain identifiable despite substantial displacement of the nerve, limiting the reliability of its usual distance or directional relationship.

Mobile cartilaginous, muscular, and vascular structures may be displaced by tumour growth, retraction, or scarring, whereas fixed osseous landmarks are generally more resistant to positional change. This mechanical distinction provides an anatomical rationale for considering preserved bony references when soft-tissue planes are altered; it does not demonstrate superior identification accuracy or nerve preservation in those settings.

### 4.4. Reproducibility and Clinical Applicability

Measurement reproducibility concerns agreement when observers repeat the same defined measurement. A small standard deviation within one series describes the spread of its observations. Between-study reproducibility also requires comparable reference points, neural endpoints, specimen preparation and measurement direction.

Clinical applicability therefore extends beyond reproducibility of distance measurements. A useful landmark should be recognisable within the operative field, contribute meaningfully to localisation of the nerve, and retain value across the circumstances in which it is intended to be used. Evidence of successful identification should be distinguished from comparative evidence concerning identification time, nerve manipulation or postoperative facial function [19,27].

The functional comparison identifies whether each judgment is supported by cadaveric, intraoperative or radiological evidence, or by anatomical interpretation.

### 4.5. Combined Landmark Strategy

Complementary landmarks can contribute different information during progressive localisation: an accessible structure establishes regional orientation, while a deeper or more fixed reference helps refine the expected position of the nerve. Published combined approaches illustrate the feasibility of integrating such information, but do not establish a universally optimal sequence or prove improved nerve preservation [10,27].

## 5. Anatomical Classification of Surgical Landmarks for Facial Nerve Identification

The following sections distinguish direct trunk measurements from distances to a branch, distances to a muscular entry point and lengths measured along a nerve. Table S1 records these endpoints separately.

### 5.1. Osseous Landmarks

#### 5.1.1. Tympanomastoid Suture

The TMS is among the most extensively investigated osseous landmarks for localisation of the main facial nerve trunk during parotidectomy. Several investigations report close relationships with limited within-study variability, although larger distances in other series preclude a universal numerical estimate. Anatomical proximity must also be distinguished from accessibility, because the TMS is often identified by palpation rather than direct visualisation.

##### Anatomical Relationship with the Facial Nerve

De Ru et al. examined the relationship between commonly used surgical landmarks and the main facial nerve trunk in 30 cadaveric hemifaces. Two observers reported mean TMS–trunk distances of 2.7 mm (SD 0.7) and 2.6 mm (SD 0.8), respectively, with 29 observations per observer. These observer-specific measurements are separate assessments of the anatomical sample, rather than independent specimens [7].

Witt et al. subsequently evaluated the TMS in a prospective study involving 14 cadaveric specimens and 22 patients undergoing parotidectomy. The shortest distance from the TMS to the facial nerve was compared with that from the posterior-superior margin of the posterior belly of the digastric muscle. In cadavers, the mean TMS–facial nerve distance was 1.8 mm (range, 0–4 mm), compared with 12.4 mm (range, 7–17 mm) for the posterior belly of the digastric muscle. Corresponding intraoperative measurements were 2.0 mm (range, 0–4 mm) and 10.7 mm (range, 5–14 mm), respectively [8].

Rea et al. investigated 26 embalmed, formaldehyde-fixed cadavers and reported TMS–trunk separation of 2.5 ± 0.4 mm, compared with 5.5 ± 2.1 mm for the posterior belly of the digastric muscle, 6.9 ± 1.8 mm for the tragal pointer and 10.9 ± 1.7 mm for the bony–cartilaginous canal junction. These findings favour the TMS for measured proximity within that study; they do not establish superior postoperative outcomes [9].

##### Intraoperative Identification and Clinical Applicability

Saha et al. investigated the TMS, tragal pointer, and posterior belly of the digastric muscle in 10 fresh cadaveric dissections and 10 live parotidectomies. The mean TMS–facial nerve distance was 3.5 mm in cadavers and 3.87 mm during live surgery [10]. However, discrepancies between some summary values and individual observations in the original report limit the precision with which these estimates can be interpreted; these inconsistencies are documented in Supplementary Table S1.

The TMS remained the closest of the three landmarks in both components of the study. By comparison, the mean distance from the facial nerve to the tragal pointer was 16.61 mm in cadavers and 16.36 mm intraoperatively, whereas the distance to the posterior belly of the digastric muscle was 7.41 mm in the cadaveric abstract (7.5 mm in Results) and 8.03 mm intraoperatively [10].

Importantly, anatomical proximity did not correspond directly to ease of use. The TMS was described as a hard ridge palpable deep to the cartilaginous external auditory canal and was more difficult to visualize directly during live surgery. The posterior belly of the digastric muscle was easier to identify and was therefore considered useful for initial orientation, with the tragal pointer and TMS providing supplementary localization [10].

Kaushal et al. provided additional clinical evidence in a series of 60 patients undergoing parotid surgery. The mean TMS–trunk distance was 2.92 ± 0.60 mm, compared with 18.38 ± 6.85 mm for the tragal pointer. The reported statistical comparison concerned the distances; the smaller dispersion around the TMS measurement should not be described as a separately demonstrated difference in variance [11].

The TMS was identified by palpation rather than by complete direct exposure, and the facial nerve was successfully located in all 60 patients [11]. Recurrent disease and pre-existing facial weakness were excluded, and the study did not independently compare postoperative outcomes attributable to landmark selection. Its findings therefore support feasibility in the examined population rather than a reduction in nerve injury or validated applicability to revision surgery.

##### Composite Use with the Mastoid Tip

Verma et al. investigated an imaginary line joining the TMS and mastoid tip. The study describes 14 hemifaces from ten fresh-frozen cadaveric heads, whereas the abstract reports 12 hemifaces. The undivided trunk crossed the line in 12 of the 14 dissections; in two, division occurred before the line was reached.

The cadaveric results in the cited study report a TMS–nerve mean distance of 5 mm, whereas its tabulated results report approximately 10.43 mm. These incompatible values should remain identified as source inconsistencies rather than be resolved by selecting the more favourable estimate.

In the five-patient clinical component, the reported TMS–nerve distance was 7.2 mm (range, 4–10 mm), and the trunk was readily identified using the line in four patients. The original article describes 4/5 as 90%; the correct proportion is 80%. This small feasibility series does not establish comparative safety, and its composite geometry is not interchangeable with shortest-distance measurements from an isolated TMS reference point [35].

Overall, the TMS is a useful deep osseous reference, but the frequently quoted range of approximately 1.8–3.87 mm does not encompass all reports. Pather and Osman reported a mean of 10.0 mm, while Shah et al. reported means of 6.50 mm in cadavers and 6.35 mm intraoperatively. These findings preclude treating a restricted range as a universal operative coordinate [27,36].

Two additional operative series place the suture–trunk relationship in clinical context. Mendoza et al. recorded a mean distance of 6.1 mm (SD 2.0, range 4–10) in 22 patients, using the anterior point of the palpable V-shaped sulcus before tumour excision [37]. Sardar et al. measured a mean of 7.58 mm (SD 1.33) in the 25 patients in whom the suture could be exposed [38].

#### 5.1.2. Mastoid Tip

##### Anatomical Basis and Operative Orientation

The mastoid tip is a fixed and readily palpable osseous structure that provides regional orientation toward the stylomastoid compartment during parotid surgery. Its surgical relevance is strengthened by its close relationship with the attachment of the posterior belly of the digastric muscle, which originates from the mastoid notch medial to the mastoid process [13,39].

The mastoid tip primarily provides regional orientation; its relationship to a displaced nerve must be reassessed in altered anatomy (Section 6).

##### Quantitative Relationship with the Facial Nerve Trunk

Stankevicius and Suchomlinov dissected 22 hemifaces from 11 donors, excluding two double-trunk specimens from morphometric analysis. Their tabulated results report a mastoid tip–trunk distance of 12.53 ± 2.30 mm (range, 8.99–17.26 mm) in the remaining 20 sides; the abstract gives 12.52 mm. The measured-side denominator and table value are retained here [33].

Verma et al.’s TMS–mastoid line addresses a different geometrical relationship. Its crossing measurements should therefore not be combined with isolated mastoid tip–trunk distances [35].

#### 5.1.3. Stylomastoid Foramen

##### Anatomical Position and Operative Orientation

The stylomastoid foramen (SMF) represents the fixed osseous exit of the facial nerve from the temporal bone and therefore defines the most proximal extracranial point of the nerve. It is located between the mastoid and styloid processes on the inferior aspect of the temporal bone [13].

The SMF defines the nerve’s osseous exit, but its depth and confined neurovascular surroundings limit its use for initial exposure.

##### Morphometry of the Facial Nerve at the Stylomastoid Foramen

Salame et al. performed bilateral microsurgical dissections of 23 formalin-fixed human cadavers and reported a mean facial nerve diameter of 2.66 ± 0.55 mm at its emergence from the SMF [40]. Hewapathirana et al. subsequently reported a smaller mean facial nerve diameter of 1.78 ± 0.31 mm at the SMF in a separate cadaveric investigation [41]. More recently, Martínez Pascual et al. examined 38 facial nerves from 23 adult cadavers and reported a mean diameter of 2.16 ± 0.49 mm in this region [42].

These are measurements of the nerve itself, not distances from an independent landmark. They should not be incorporated into comparisons of landmark–trunk separation or interpreted as operative safety margins.

#### 5.1.4. Styloid Process

##### Anatomical Position and Operative Orientation

The styloid process is a slender projection of the temporal bone situated anteromedial to the mastoid process. Its length and angulation vary between individuals [43]. Its base, considered together with the mastoid origin of the posterior belly of the digastric muscle, provides a regional reference for facial nerve localisation. This composite relationship should be distinguished from measurements involving the distal styloid tip [44].

##### Quantitative Relationship with the Facial Nerve Trunk

Pather and Osman performed microdissection of 40 adult cadavers and measured the shortest distance between the facial nerve trunk and several anatomical landmarks. The distance between the styloid process and the facial nerve ranged from 4.3 to 18.6 mm, with a mean of 9.8 mm [36]. This value was subsequently included in the systematic review by Ji et al. but represents a single contributing study rather than a pooled styloid–nerve estimate [12].

Joseph et al. investigated a composite relationship between the styloid base and mastoid origin of the posterior belly of the digastric muscle. The reported mean separation of 0.72 cm (range, 0.45–0.99 cm) describes the two reference structures, not a styloid–facial nerve distance. The accompanying 25-patient clinical series demonstrated identification feasibility without an independent comparative test of nerve preservation [44].

#### 5.1.5. External Auditory Canal and Tympanic Plate

##### Anatomical Basis and Operative Orientation

The external auditory canal consists of a lateral cartilaginous and medial osseous component and represents an important regional structure during parotid surgery [45,46]. These components should not receive a single assessment of resistance to distortion: the bony canal provides a relatively rigid reference, whereas the cartilaginous canal can deform under traction.

Variation in tympanic-plate morphology [47,48,49] and intratemporal facial nerve–annulus relationships [50,51] provides background anatomy rather than direct evidence for extracranial trunk localisation.

##### Relationship with the Facial Nerve

Pather and Osman [36] reported a mean distance of 13.4 mm between the facial nerve trunk and the external auditory canal in their cadaveric series. Rea et al. [9] specifically assessed the junction between the bony and cartilaginous external auditory canal and reported a mean distance of 10.9 ± 1.7 mm from the facial nerve trunk. These broadly named canal and precisely defined junctional references should not be assumed to represent identical measurement origins.

### 5.2. Cartilaginous Landmarks

#### Tragal Pointer

The tragal pointer is among the most frequently used superficial landmarks for anterograde facial nerve identification. Its principal advantage is accessibility: the tragal cartilage is encountered early during conventional parotid exposure and can provide immediate orientation toward the deeper facial nerve trunk [27,52].

##### Anatomical Basis and Operative Orientation

The tragal pointer represents the medial projecting end of the tragal cartilage. During parotidectomy, dissection can be continued along the tragal cartilage toward its deep end, after which the facial nerve is generally sought deeper and inferior to the cartilaginous reference [52].

The term “pointer” should not be interpreted as implying a fixed vector toward the facial nerve. The cartilage varies in length, shape, orientation, and rigidity, and can also be displaced by traction during surgical exposure [36,52].

##### Quantitative Relationship with the Facial Nerve Trunk

Pather and Osman [36] reported a mean distance of 34.0 mm between the tragal pointer and facial nerve trunk, with values ranging from 24.3 to 49.2 mm in 40 adult cadavers. Rea et al. [9], in contrast, reported a mean distance of only 6.9 ± 1.8 mm in their investigation of 26 embalmed cadavers.

The cause of this discrepancy cannot be established from the numerical findings alone. Pather and Osman describe microdissection and shortest-distance measurements, while Rea et al. [9] explicitly describe embalmed, formaldehyde-fixed specimens. The available primary-source descriptions do not establish that both studies used an identical cartilaginous reference point or measurement trajectory. Differences in reference-point definition, preparation and positioning are therefore plausible contributors, rather than demonstrated explanations [9,36].

Within Shah et al.’s combined investigation of 41 cadavers and 20 patients, reported tragal pointer–trunk distances were 13.04 ± 5.238 mm in 82 cadaveric regions and 9.95 ± 3.967 mm intraoperatively (*p* = 0.036). This within-study difference supports caution when transferring measurements between settings, but does not identify preservation, retraction or any other individual factor as its cause [27].

##### Surgical Utility and Multi-Landmark Use

**Figure 3:**
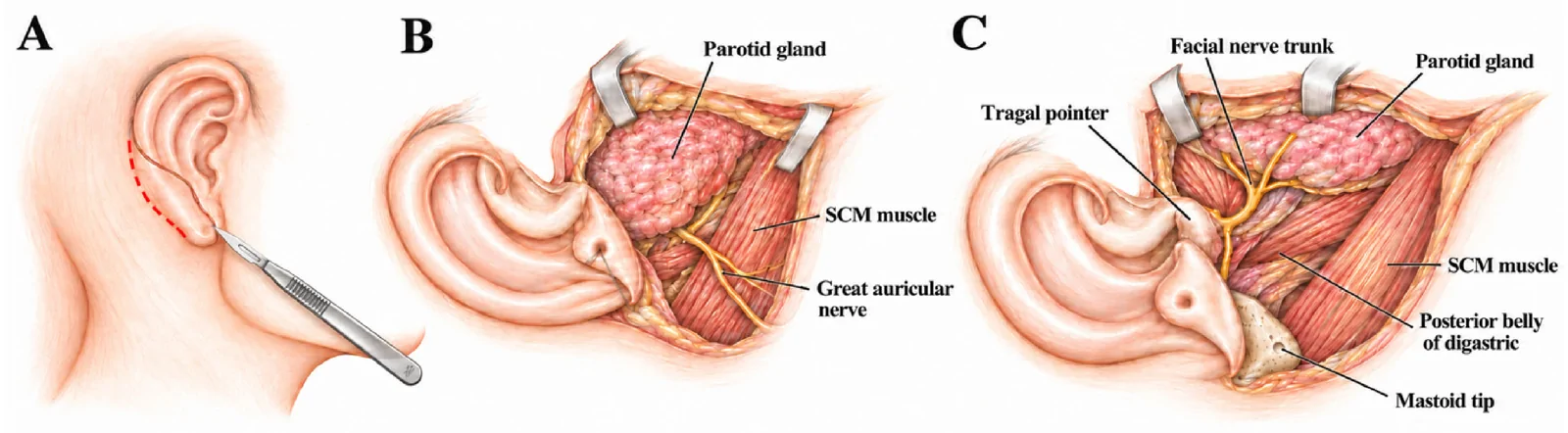
Surgical exposure and anatomical landmarks for identification of the facial nerve trunk during parotidectomy. (**A**) Preauricular and retroauricular incision planning, with the intended incision indicated by a red dashed line. (**B**) Exposure of the parotid gland following skin-flap elevation, demonstrating its relationship with the sternocleidomastoid muscle and great auricular nerve. (**C**) Deeper surgical exposure illustrating the tragal pointer, mastoid tip, posterior belly of the digastric muscle, and main facial nerve trunk. These structures provide complementary anatomical references for progressive localisation of the facial nerve during anterograde parotid dissection.

Despite limited precision as an isolated landmark, the tragal pointer remains useful because it is encountered early and provides superficial orientation toward the facial nerve. Shah et al. [27] incorporated it as the first component of the cartilage–muscle–suture (C–M–S) technique. In this sequence, the tragal pointer is identified first (Figure 3), followed by the posterior belly of the digastric muscle and then the tympanomastoid suture.

Successful trunk identification in the combined series cannot be attributed to the tragal pointer independently or equated with reduced postoperative dysfunction. Its role is early orientation within a published combined technique, rather than provision of a fixed nerve coordinate.

### 5.3. Muscular Landmarks

#### 5.3.1. Posterior Belly of the Digastric Muscle

The posterior belly of the digastric muscle (PBDM) is one of the most frequently used muscular landmarks for anterograde identification of the facial nerve trunk. It originates from the mastoid notch and courses anteroinferiorly toward the intermediate tendon, forming a readily recognisable structure along the inferior aspect of the parotid region [10,16].

##### Quantitative Relationship with the Facial Nerve Trunk

Published measurements use different portions of the muscle as their starting point. Witt et al. [8] measured from its posterior-superior margin, reporting means of 12.4 mm in cadaveric specimens and 10.7 mm intraoperatively. Pather and Osman [36] measured from the muscle attachment and reported a mean of 14.6 mm. These definitions should not be treated as interchangeable.

Rea et al. [9] reported a considerably shorter mean distance of 5.5 ± 2.1 mm. Saha et al. measured from the mastoid attachment, termed the “insertion” in their report. Their cadaveric mean is given as 7.41 mm in the abstract and 7.5 mm in the Results, whereas the intraoperative mean is 8.03 mm. The discrepancy is retained in Supplementary Table S1 rather than silently harmonised [10].

Singh et al. [34] retrospectively evaluated 24 superficial parotidectomies and reported a mean PBDM–trunk distance of 17 ± 0.87 mm, with an observed range of 15–20 mm.

Differences between attachment, border and closest-point measurements plausibly contribute to the spread of results, but their relative contribution has not been established. Neither a larger mean distance nor agreement within a selected series independently demonstrates greater clinical reliability.

##### Surgical Accessibility and Clinical Utility

The principal strength of the PBDM lies in its intraoperative recognisability. Saha et al. [10] found it to be the most easily identifiable of the three landmarks assessed during live parotidectomy and recommended it as a useful initial reference before supplementary localisation with the tragal pointer or tympanomastoid suture.

Singh et al.’s series concerned 24 patients with superficial-lobe pleomorphic adenomas and excluded redo surgery, deep-lobe involvement and preoperative facial nerve involvement. No permanent facial nerve injuries were reported, but the uncontrolled design, small sample and limited outcome characterisation do not establish superiority over alternative landmarks or applicability to revision surgery [34].

When tumour displacement or fibrosis alters the muscle’s relationships, additional preserved references may assist reassessment. The PBDM should therefore be regarded as an accessible regional guide, without assuming a constant millimetric relationship or a universally appropriate position within a fixed operative sequence.

#### 5.3.2. Stylohyoid Muscle

##### Anatomical Position and Operative Orientation

The stylohyoid is a slender suprahyoid muscle extending from the styloid process toward the hyoid region and lies in close relationship with the posterior belly of the digastric muscle [53]. It is supplied by a branch of the facial nerve arising from the proximal extracranial trunk [40].

Its relevance is primarily topographical; the available evidence does not establish it as an independently validated predictor of trunk position.

##### Anatomical Variation and Surgical Limitations

Ozgur et al. [54] examined 56 sides from 28 cadavers and identified atypical attachments of the stylohyoid muscle in 22 sides, including bilateral atypical morphology in ten specimens.

These findings demonstrate variation in the muscular complex, but do not directly measure its accuracy for facial nerve localisation. Similarly, variation around the digastric intermediate tendon concerns more distal muscular anatomy and should not be presented as direct evidence of altered proximal nerve position. The stylohyoid is consequently a supplementary regional reference rather than an established primary localisation landmark.

### 5.4. Vascular Landmarks

#### 5.4.1. Retromandibular Vein

##### Anatomical Position and Operative Orientation

The retromandibular vein (RMV) is formed within the parotid gland by the union of the superficial temporal and maxillary veins. In the conventional arrangement, the facial nerve and its divisions lie superficial to the RMV, while the external carotid artery occupies a deeper plane [55].

This relationship has made the RMV an important surgical and radiological reference for the facial nerve plane. However, the relationship is not invariant. The vein may lie lateral to portions of the nerve, pass between facial nerve divisions, or demonstrate more complex configurations [55,56].

##### Anatomical Variation and Clinical Utility

**Figure 4:**
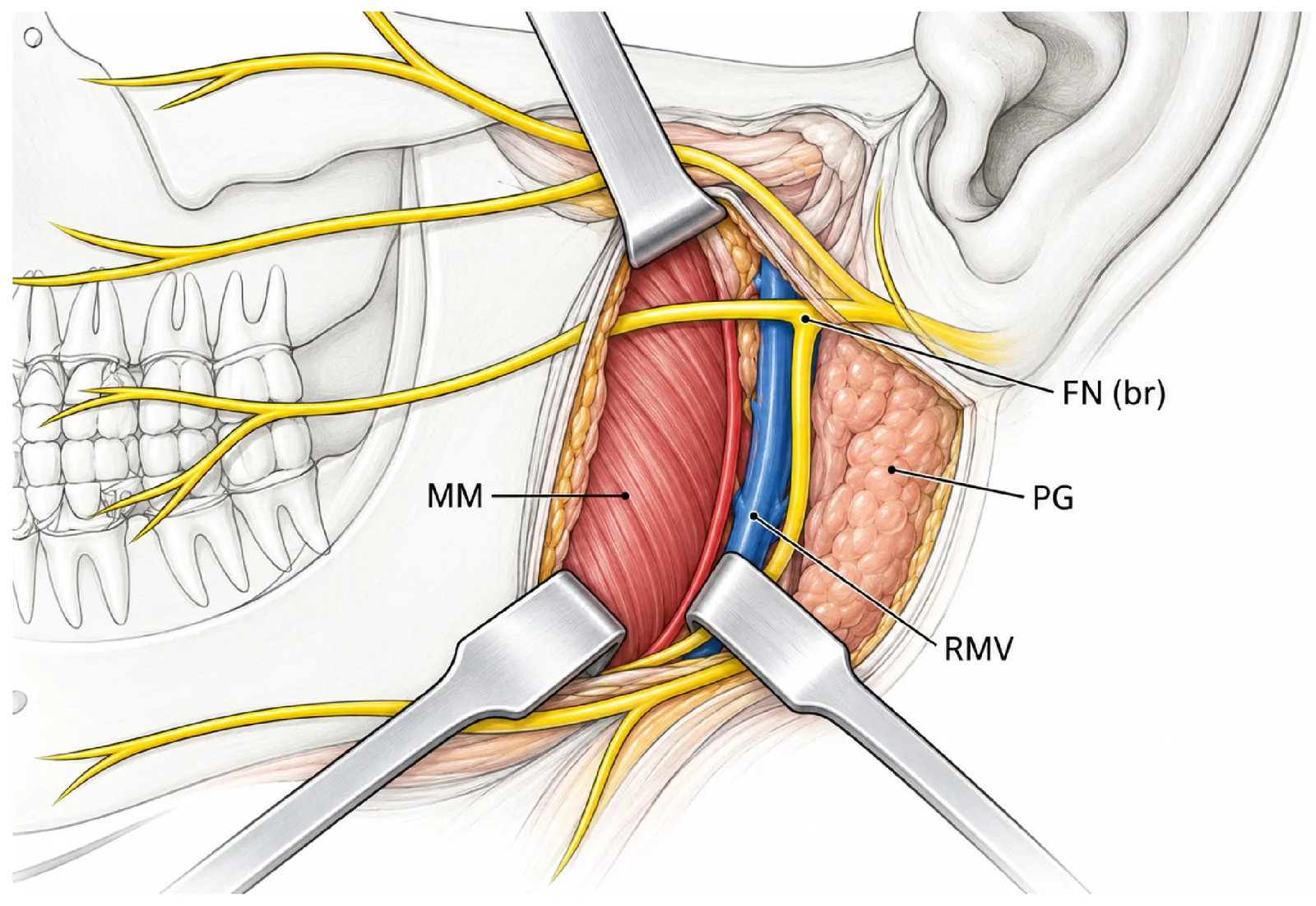
Anatomical relationship between the facial nerve and retromandibular vein in the parotid region. Schematic illustration demonstrating the course of the facial nerve (FN) and its branches in relation to the retromandibular vein (RMV), parotid gland (PG), and masseter muscle (MM). The depicted configuration does not encompass all reported neurovascular variants.

In an anatomical study of 132 parotid glands, Touré and Vacher [56] found the RMV medial to the facial nerve in 65.2% of cases and lateral to the nerve in 13%. Additional configurations included cases in which the nerve lay between superficial and deep venous planes. Poutoglidis et al. [55] subsequently identified three principal patterns in 24 cadaveric hemifaces. Figure 4 illustrates one neurovascular configuration.

These variants limit use of the RMV as an invariant surrogate for the nerve and can complicate haemostasis near neural structures.

Zhong and Ashwell [57] measured the distance from the highest point of the RMV below the parotid gland to the marginal mandibular branch after its exit from the gland. The mean (SD) distance was 10.2 (5.6) mm in 23 male specimens and 12.4 (7.1) mm in seven female specimens. These are distal branch measurements, not distances to the main trunk.

#### 5.4.2. External Carotid Artery

##### Anatomical Position and Operative Orientation

The external carotid artery (ECA) enters the deep aspect of the parotid gland and ultimately divides into the superficial temporal and maxillary arteries. In the conventional parotid arrangement, the facial nerve occupies the most superficial plane, the RMV an intermediate plane, and the ECA the deepest plane [55].

Variation in the cervical branches of the ECA is relevant when exposure extends into the neck or requires vascular control. Studies of facial-artery variants and common linguofacial origins provide this regional context [58,59], but do not establish landmarks for intraparotid trunk localisation.

The ECA primarily informs deep-compartment orientation. Radiological assessment of vascular surrogates predicts a lesion’s relationship to an inferred nerve plane; it does not directly visualise or surgically identify the trunk [60]. The available evidence does not establish a sufficiently invariant ECA–trunk distance for use as a precise primary landmark.

#### 5.4.3. Posterior Auricular, Stylomastoid, and Occipital Arteries

##### Anatomical Basis

Arterial structures surrounding the stylomastoid foramen (SMF) provide another potential route for identification of the proximal facial nerve. The stylomastoid artery (SMA) accompanies and supplies the facial nerve in the stylomastoid region. Its origin is variable, arising most commonly from either the posterior auricular artery (PAA) or occipital artery (OA) [61,62].

Their proximity provides an anatomical rationale for supplementary orientation, but also creates a potential source of bleeding adjacent to the nerve.

##### Anatomical Evidence

Upile et al. [61] evaluated the SMA in both cadaveric material and a clinical series of routine parotidectomies. This provides feasibility evidence for arterial guidance, without establishing an independent reduction in nerve injury.

Liu et al. [63] specifically investigated the PAA and reported that the facial nerve trunk crossed the artery inferior to the SMF in all ten dissected sides.

Reddy et al. [64] similarly evaluated 14 cadaveric hemifaces and reported an average PAA–facial nerve trunk distance of approximately 7 mm, with a range of 2–14 mm. The accessible abstract does not identify this value as a median. Possible cohort overlap with the subsequent Verma et al. [35] report has not been resolved, so the publications should not automatically be regarded as independent anatomical replications.

More recent anatomical evidence was provided by Wen et al. [62] who dissected 20 sides from ten fresh cadaveric heads. The PAA arose from the ECA in 19 specimens and from the OA in one. It passed lateral to the facial nerve in 15 cases, medial in three, and penetrated the nerve in two. The SMA originated from the PAA in 17 cases and from the OA in three and was medial to the facial nerve in 16 cases and lateral in four. The OA maintained a generally posterior and inferior relationship to the nerve, with a mean FN–OA distance of 4.99 ± 1.09 mm.

These findings demonstrate close but variable arterial relationships. Wen et al.’s specimens underwent arterial silicone perfusion, and the study did not validate clinical performance in tumour-distorted or revision fields. Arterial references should therefore remain supplementary, with each reported measurement interpreted according to its actual vascular and neural endpoints.

### 5.5. Neural Landmarks

#### 5.5.1. Great Auricular Nerve

##### Anatomical Position and Operative Role

The great auricular nerve (GAN) is a superficial sensory branch of the cervical plexus arising from the C2 and C3 anterior rami. It emerges from the posterior border of the sternocleidomastoid muscle and ascends superficially toward the parotid region and auricle, where it divides into variable terminal branches [65,66].

Because of this superficial course, the GAN is readily encountered during elevation of the parotid flap and provides useful orientation to the sternocleidomastoid and parotid region. It is not, however, directly continuous with the facial nerve and should therefore not be regarded as a primary neural landmark for tracing the facial nerve trunk.

##### Anatomical Variation and Relationship to Facial Nerve Size

Kriengkraikasem et al. [67] measured 31 sides from 16 cadavers. GAN width before division and main facial nerve trunk width before bifurcation averaged 3.26 mm (SD 0.67) and 3.36 mm (SD 0.71), respectively. Their correlation describes nerve size, not a route for locating the trunk.

#### 5.5.2. Posterior Auricular Nerve

##### Anatomical Basis

The posterior auricular nerve (PAN) is one of the first extracranial branches of the facial nerve and arises near the SMF. Unlike the GAN, the PAN is directly continuous with the facial nerve trunk and can therefore theoretically be followed proximally toward it [68,69].

Smith and Ross [68] dissected 11 facial sides from six cadavers and found that the PAN arose from the posterolateral aspect of the facial nerve trunk at a mean distance of 5.4 ± 3.3 mm from the SMF, with a range of 1.6–11.1 mm. The nerve arose as a single branch in 45.4% of specimens, while common trunks dividing into two or three branches were also observed.

##### Surgical Potential

Blau et al. [69] subsequently investigated the PAN in 75 cadaveric heminecks. The posterior auricular muscle, artery, and nerve were identified in all specimens, and the PAN could be traced toward the facial nerve trunk. The mean nerve length from the auricularis posterior muscle to the facial nerve trunk was 28 ± 6.2 mm, and the mean angle between the PAN and vertical facial nerve trunk was 39.5 ± 7.7°. The length describes the nerve’s course between structures rather than a shortest straight-line separation.

Proposed usefulness in revision surgery rests on the possibility that a posterior route remains less disturbed than previously operated preauricular tissue. This is a context-dependent anatomical rationale, not demonstrated resistance to scarring or evidence of superior safety in revision patients. The PAN is therefore a potential alternative tracing route whose applicability depends on preserved anatomy and clinical validation.

#### 5.5.3. Digastric Branch of the Facial Nerve

The digastric branch of the facial nerve (DBFN) arises from the proximal extracranial facial nerve and supplies the posterior belly of the digastric muscle. Its direct continuity with the main trunk provides an anatomical basis for proximal tracing [16].

Tayebi Meybodi et al. [16] examined 15 sides from eight cadaveric heads and identified the DBFN in all specimens. The branch originated from the facial nerve outside the SMF and had a mean length of 15.4 ± 3.4 mm. In every specimen, it inserted on the superomedial aspect of the posterior belly of the digastric muscle.

The distance between the mastoid tip and the point at which the DBFN entered the posterior belly of the digastric muscle averaged 13.6 ± 2.0 mm, with a range of 10–17 mm [16]. This endpoint is the branch’s muscular entry point, not the main facial nerve trunk, and should not be compared directly with muscle–trunk separation.

DBFN tracing therefore remains a supplementary anatomical option requiring clinical evaluation in previously treated fields.

### 5.6. Integrative Interpretation of Landmark Categories

Landmark categories provide different forms of information: osseous structures offer regional or deep references; cartilage and muscle may facilitate early orientation; vessels define variable neurovascular relationships; and branches arising from the facial nerve provide anatomical continuity towards the trunk.

These complementary rolesinform the functional comparison and the operative framework in Section 8. Applicability in altered anatomy depends on the structures and tissue planes that remain preserved.

## 6. Factors Affecting Landmark Reliability

Tumour growth, previous surgery, fibrosis and radiotherapy may displace the nerve or alter the tissue planes needed for identification. The following sections distinguish evidence from benign, malignant and previously treated fields.

### 6.1. Tumour-Related Distortion

Tumour size and location may alter the relationship between the facial nerve, parotid parenchyma and surrounding landmarks. Evidence concerning displacement by benign tumours should be distinguished from malignant disease, in which direct neural involvement may also affect the feasibility of nerve preservation. In a clinical study of parotid carcinoma, facial nerve invasion was defined by gross intraoperative infiltration with pathological confirmation, illustrating an outcome distinct from displacement or tumour–nerve contact alone [70].

Sardar et al. used spring callipers during surgery for 32 benign superficial-lobe masses. Enrolment required identification of at least two landmarks. Correlations between tumour volume and pointer-, muscle- or suture-to-trunk distance were small (r = −0.11, 0.04 and −0.16, respectively) and none reached statistical significance [38]. The series provides direct accessibility observations in selected superficial tumours. It cannot establish stability in large deep-lobe lesions.

A tumour may displace the nerve while leaving the tympanomastoid suture or mastoid process stationary. Structural stability of a bony landmark therefore does not establish stability of its relationship to the nerve.

### 6.2. Revision Surgery

Revision parotidectomy represents one of the most challenging settings for landmark-based facial nerve identification. Previous surgery may produce dense fibrosis, loss of normal tissue planes, and adherence between the facial nerve and surrounding structures. Landmarks that were readily identifiable during primary surgery may therefore become obscured, while the nerve itself may no longer be separated from adjacent tissues by its usual dissection planes [71].

Kucharska et al. studied 72 patients who underwent 109 revision procedures for mixed benign and malignant indications. At least two years after the final revision, the authors report House–Brackmann grade I in 40/72 patients (55.6%) and grades II–VI in 32/72 (44.4%). These are final grades, including pre-existing weakness. The article also reports worsening relative to preoperative grade as 27/73 (37%), although the cohort contained 72 patients. A patient-level deterioration rate with a consistent denominator cannot be recovered from those figures [71].

A preserved peripheral branch may offer an alternative route when scar prevents proximal identification, but the revision series does not compare the safety of anterograde and retrograde dissection.

### 6.3. Fibrosis, Previous Radiotherapy, and Inflammatory Change

Inflammatory fibrosis and previous radiotherapy should be considered separately because their clinical evidence is not interchangeable. Fibrosis may obscure the tissue interfaces needed to progress from an identifiable landmark to the nerve. Thus, a landmark may remain recognisable while the intervening dissection plane becomes difficult to develop.

Sharma et al. [72] retrospectively described 39 superficial parotidectomies: 29 underwent conventional proximal exploration, whereas 10 underwent distal exploration of the buccal branch because post-inflammatory fibrosis made main-trunk identification difficult. This supports the feasibility of an alternative route in selected cases. Because the approach was selected according to operative difficulty, the groups were not directly comparable, and the study does not demonstrate superior facial nerve safety with distal exploration.

Previous radiotherapy was discussed as another potential cause of difficult proximal identification, but these findings do not constitute a dedicated comparison in an irradiated population [72]. Recommendations for irradiated fields therefore remain conditional. Landmark selection and dissection direction should reflect the anatomy and tissue planes actually preserved, rather than an assumed hierarchy established in untreated specimens.

## 7. Evidence-Based Comparison of Surgical Landmarks

Two operative observations illustrate why the functional comparison compares landmarks by function. Saha et al. found the posterior belly of digastric easiest to recognise during surgery, although the suture was closest to the trunk [10]. In Sardar et al., the pointer was exposed in 32/32 operations, the muscle in 26/32 and the suture in 25/32 [38]. Entry into that series required successful identification of at least two landmarks. The fractions describe those selected benign superficial-lobe cases.

Table 1 compares early orientation, deep localisation and neural tracing, while identifying the evidence supporting each role.

## 8. Surgical Decision-Making and Operative Framework

**Figure 5:**
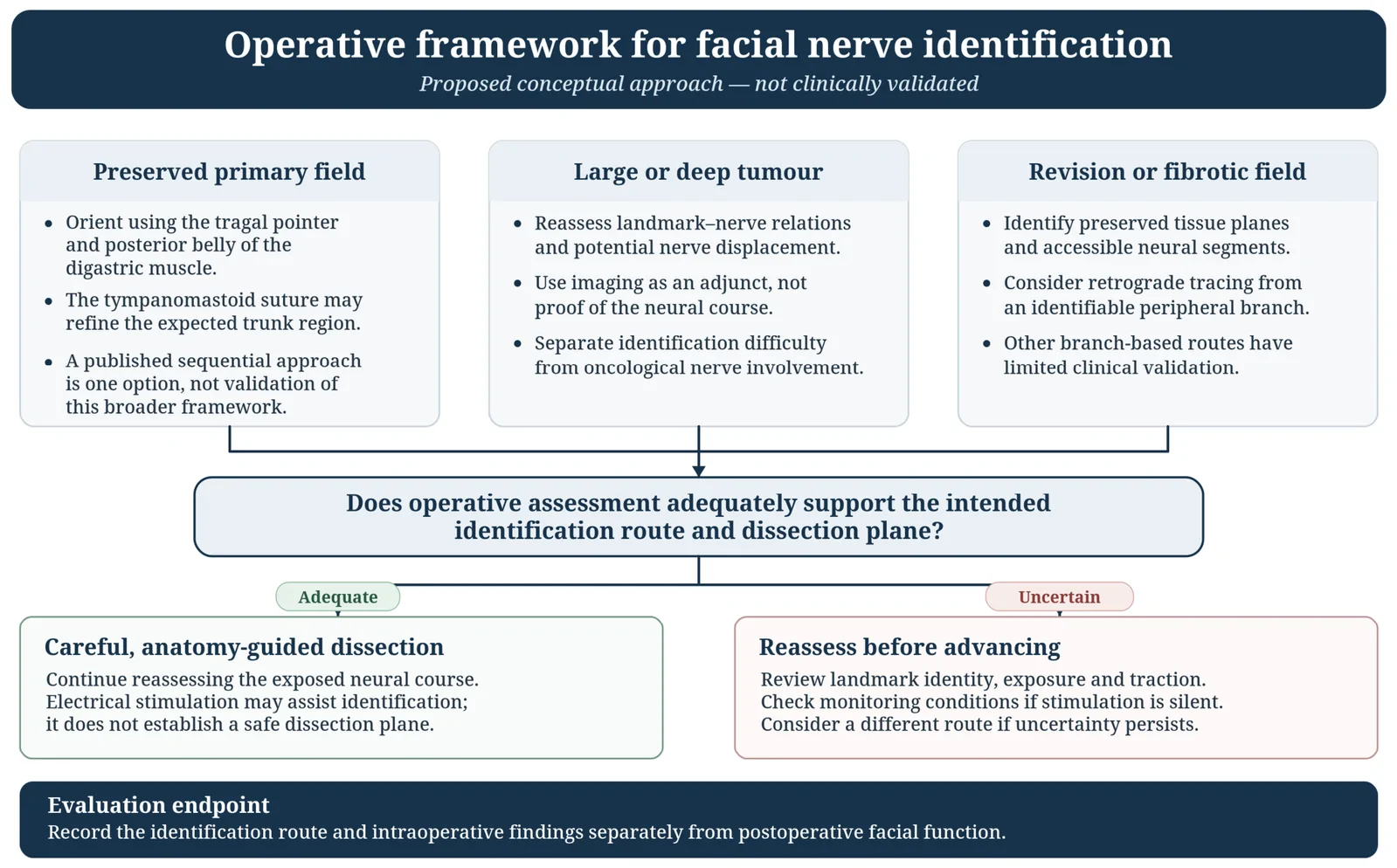
Unvalidated operative framework for facial nerve identification. The authors’ unvalidated framework adapts identification routes to preserved, tumour-distorted, or revision and fibrotic anatomy; these contexts may overlap. Concordant landmarks do not confirm neural identity or a safe plane. Imaging and neuromonitoring are adjuncts, and an absent evoked response does not exclude neural tissue.

The practical objective of landmark-based facial nerve identification is not to determine which structure is universally “best”, but to progressively reduce uncertainty regarding the position of the nerve while minimising unnecessary dissection. The following framework organises anatomical observations and selected clinical findings into a proposed approach to operative reasoning. The framework in Figure 5 remains unvalidated.

### 8.1. Routine Primary Parotidectomy

When anatomy is preserved, initial orientation can be obtained using readily accessible structures such as the tragal cartilage, mastoid region, and posterior belly of the digastric muscle. These structures define the general operative corridor but should not be interpreted as determining an exact location of the nerve.

A deeper reference, such as the tympanomastoid suture, may refine the expected trunk position. Saha et al. demonstrated how this proximity can complement the earlier accessibility of muscle and cartilage [10].

The cartilage–muscle–suture (C–M–S) technique described by Shah et al. [27] sequentially uses the tragal pointer, posterior belly of the digastric muscle and tympanomastoid suture. This published technique should be distinguished from the broader framework proposed here, which additionally incorporates pathological distortion, uncertain orientation and alternative identification routes. Successful identification using C–M–S does not independently validate these additional components.

### 8.2. Large or Deep Parotid Tumours

Large or deep tumours require reassessment of landmark–nerve relationships because the nerve may be displaced relative to stationary bone (Section 6.1).

Imaging may supplement this assessment. Retromandibular-vein-based ultrasound estimates the nerve plane indirectly, whereas dedicated MR neurography attempts to depict the nerve itself [73,74,75]. Neither approach eliminates the need to reconcile preoperative predictions with the anatomy encountered during surgery.

Malignant disease also requires distinction between difficulty identifying the nerve and tumour involvement that limits its preservation. Decisions concerning oncological resection cannot be determined by landmark proximity or imaging visibility alone.

### 8.3. Revision, Fibrotic, or Previously Treated Fields

When scarring obscures the proximal corridor, operative planning should consider which tissue planes and neural segments remain identifiable. A preserved peripheral branch may permit retrograde tracing when proximal identification is difficult. This is a context-dependent anatomical rationale, rather than evidence that retrograde dissection is inherently safer in scarred fields.

The comparisons in Section 3.2 and Table S2 chiefly concern benign surgery. In particular, the Mashrah meta-analysis excluded revision, recurrent and malignant disease [30]; its findings cannot establish a safety advantage in these populations.

Distal buccal-branch exploration was feasible in the selected fibrotic cases described by Sharma et al. [72], but allocation according to operative difficulty prevents a reliable comparison of injury risk (Section 6.3).

Posterior auricular nerve or digastric-branch tracing offers additional anatomical possibilities when those branches remain identifiable. Their proposed value in difficult fields is supported predominantly by cadaveric investigations, with limited comparative clinical validation [16,68,69].

### 8.4. Discordant Landmarks

Within this framework, “discordant landmarks” refers to independently identified structures suggesting incompatible nerve regions or trajectories after accounting for their defined reference points, the three-dimensional operative view and tissue displacement. No validated numerical threshold or concordance score is proposed.

Discordance should prompt reassessment of landmark identity, exposure, retraction and the available dissection plane. Imaging or functional stimulation may provide additional information, but neither a familiar landmark nor a positive response establishes that the next dissection step is safe.

For reproducible evaluation, future studies could predefine landmark points and patient positioning, record the nerve regions predicted independently by each landmark, and compare these predictions with direct operative findings. Relevant outcomes would include localisation error, observer agreement, identification time and postoperative facial function.

### 8.5. Proposed Operative Framework

Intermittent electrical stimulation and monitoring during dissection serve related but distinct purposes. Stimulation of suspected neural tissue tests for an evoked response in the monitored facial muscles and may support identification when interpreted alongside the anatomy. Free-running electromyography provides feedback during subsequent manipulation, including mechanically evoked activity; it does not continuously demonstrate intact conduction along every facial nerve branch.

An absent evoked response does not exclude neural tissue. Interpretation requires consideration of neuromuscular blockade, electrode placement and connections, equipment function, stimulation conditions, intervening tissue and pre-existing neural dysfunction. When anatomical identification remains uncertain, the appropriate response is reassessment of the operative anatomy and monitoring setup rather than treating non-responsive tissue as non-neural [76].

Functional assistance should also be distinguished from demonstrated postoperative benefit. In a prospective two-centre comparison of 100 primary parotidectomies in 96 patients, 50 procedures used EMG monitoring with visual observation and 50 used visual observation alone. Immediate and definitive facial outcomes did not differ significantly, although superficial-parotidectomy duration was shorter with monitoring [77]. This individual study does not establish equivalence or exclude benefit in other clinical settings.

## 9. Emerging Technologies and Image-Guided Localisation

Technologies used to support facial nerve identification address different outcomes: indirect prediction of the nerve plane, direct nerve visualisation, intraoperative display of reconstructed anatomy, experimental optical identification, and prediction of postoperative dysfunction. Their performance measures are therefore not interchangeable, and technical accuracy should not be interpreted as evidence of improved nerve preservation.

### 9.1. Ultrasonography

High-resolution ultrasonography can estimate tumour location and its relationship to the expected facial nerve plane using surrounding anatomical structures. In a prospective study of 50 patients with benign parotid tumours, Psychogios et al. [73] correctly classified tumour–nerve contact in 48/50 patients (96%), using operative findings as the reference standard. Contact was recorded as present or absent; no numerical distance threshold was specified. The nerve course was inferred indirectly, particularly from the retromandibular vein, rather than continuously visualised.

The predicted surgical technique agreed with the procedure performed in 49/50 patients (98%). The available categories were extracapsular dissection, partial parotidectomy with main-trunk exposure, superficial parotidectomy, and subtotal or total parotidectomy. Ultrasound findings were documented before surgery and withheld from the operating investigator. Nevertheless, agreement with the operation performed does not independently establish that the procedure was optimal. These estimates describe anatomical classification and operative planning, rather than demonstrated improvement in facial nerve preservation.

Interpretation also depends on the visibility and reliability of the surrogate structures. Anatomical variation in the relationship between the retromandibular vein and facial nerve remains relevant when applying these findings beyond the studied benign-tumour population.

### 9.2. Magnetic Resonance Neurography and Three-Dimensional Imaging

Dedicated MR sequences permit more direct assessment of the facial nerve and its relationship to parotid tumours. However, visibility of the main trunk, depiction of individual divisions, agreement with operative anatomy and postoperative function represent distinct endpoints.

Jeong et al. [74] prospectively evaluated 296 patients, including 122 who underwent three-dimensional double-echo steady-state water-excitation MRI (3D-DESS-WE-MRI). Among 120 evaluable examinations, the main trunk was visible in 117 (97.5%), and tumour location relative to the nerve agreed with surgical findings in 109 (90.8%). Following exclusion of intentional nerve sacrifice, clinical comparisons included 105 MRI and 165 non-MRI patients, with propensity methods used to address measured case differences. In the matched comparison, median nerve-identification time was 25 min with MRI and 35 min without MRI. Temporary and permanent palsy did not differ significantly; the corresponding odds ratios were 2.29 (95% CI 0.64–8.25) and 2.02 (95% CI 0.32–12.90). These wide intervals do not establish equivalence.

Gaudino et al. [75] retrospectively assessed 40 consecutive patients using two independent readers and comparison with surgical findings. Distal divisions were visible in 34% of cases. Reported localisation accuracy against surgery was 100% for the main trunk, 96% for the temporofacial division and 89% for the cervicofacial division. These localisation results are distinct from visibility rates and do not establish a reduction in postoperative nerve injury.

### 9.3. Navigation and Mixed-Reality Visualisation

Hu et al. evaluated preoperative nerve segmentation and intraoperative mixed-reality navigation in 15 patients [78]. The main trunk was visible preoperatively in all 15. Their separate assessment sampled points along the reconstructed nerve. The precise sites and spatial error accepted as correct could not be verified, which limits interpretation of the point count (Table S2). The series establishes technical feasibility. It does not quantify localisation error across the operative field or establish an improvement in postoperative facial function.

Future evaluations should explicitly define the anatomical validation points, accepted spatial error, registration method and effects of operative tissue displacement. Comparative studies are required to determine whether navigation improves identification time or postoperative facial function.

### 9.4. Fluorescence-Guided Nerve Identification

Fluorescence-guided imaging seeks to distinguish neural tissue directly from surrounding structures. Hussain et al. [79] investigated fluorescently labelled nerve-binding peptide NP41 in a murine model of parotid cancer. Twenty-eight tumour-bearing mice underwent surgery with fluorescence guidance or white-light visualisation, and additional experiments evaluated nerve contrast. Fluorescence improved the distinction between nerves and surrounding tissues, including smaller buried branches. Postoperative facial function was assessed using automated whisker tracking.

These findings provide preclinical evidence of enhanced optical identification. They do not establish efficacy in human parotidectomy. Human safety, neural specificity, imaging depth and comparative functional outcomes require separate evaluation before findings from this experimental approach can support clinical recommendations.

### 9.5. Artificial Intelligence and Predictive Modelling

Prediction of postoperative facial weakness addresses prognosis rather than intraoperative localisation. Chiesa-Estomba et al. [80] evaluated random forest, K-nearest-neighbour and artificial neural-network models using retrospective multicentre data from 736 patients undergoing surgery for benign parotid tumours. Revision procedures and cases with malignant final histology were excluded.

These models predict postoperative dysfunction affecting at least one facial nerve branch. Clinical use requires external validation, calibration and confirmation that predictors are available at the intended decision point.

## 10. Evidence Gaps and Future Research Priorities

Despite extensive anatomical literature describing facial nerve landmarks, the evidence base remains limited in several clinically important respects. As a critical narrative review, the present synthesis is also susceptible to selection and interpretative bias. Heterogeneity in study methods and populations limits direct comparison and prevents a universal ranking of landmark performance.

First, landmark definitions and measurement methods are insufficiently standardised. Differences between published measurements, particularly for the tragal pointer and posterior belly of the digastric muscle, may reflect variation in reference points, neural endpoints, specimen preparation and measurement trajectories; these explanations should be verified rather than assumed. Future studies should define landmarks precisely and describe measurement planes in a reproducible manner. Independent donors, bilateral specimens, repeated readings and patients should be reported separately, with paired observations accounted for statistically. The type of dispersion accompanying each estimate should also be specified.

Second, anatomical proximity is frequently used as a surrogate for surgical usefulness. Most studies measure distance to the facial nerve but do not determine whether a landmark is easier to identify, reduces dissection time, decreases nerve manipulation, or improves postoperative facial nerve function. Future investigations should combine anatomical measurements with clinically relevant operative outcomes. Baseline function, branch-specific weakness, follow-up intervals and the definition of persistent dysfunction require consistent reporting.

Third, difficult operative fields are underrepresented. Much of the quantitative evidence derives from normal cadavers or uncomplicated primary parotidectomy. Primary benign, deep-lobe, malignant, revision, irradiated and inflammatory populations should be evaluated separately. Neither structural stability nor performance in untreated anatomy establishes clinical reliability in previously treated fields.

Fourth, multi-landmark strategies require prospective validation. The proposed framework requires predefined landmark points, patient positioning, criteria for discordance and reasons for changing identification routes. Evaluation should compare localisation error, observer agreement, operative efficiency and postoperative function against an appropriate alternative strategy, with adjustment for case mix and surgeon experience.

Fifth, imaging studies should increasingly compare preoperative prediction with direct intraoperative anatomy. Visibility, anatomical classification, segmentation, registration and functional outcomes should remain separate endpoints. For navigation, accepted spatial error and changes caused by operative tissue displacement require explicit reporting.

Sixth, clinical studies should evaluate whether posterior auricular or digastric-branch tracing is feasible and beneficial in revision and irradiated populations, where current support remains predominantly anatomical.

Prospective multicentre studies incorporating tumour characteristics, imaging findings, landmarks used, direction of nerve dissection, use of neuromonitoring, operative time, and standardised postoperative facial nerve outcomes would provide substantially stronger evidence for future operative recommendations.

## 11. Conclusions

The tympanomastoid suture can refine the deep search for the facial nerve, but the spread of published means precludes a universal depth estimate. The tragal pointer and posterior belly of digastric offer earlier orientation when they can be exposed. The evidence for tracing small neural branches in scarred fields remains predominantly anatomical.

Selection of proximal, retrograde or alternative tracing routes should remain conditional on the anatomy encountered, pathology and planned resection. Findings from primary benign surgery should not be generalised to revision, malignant or irradiated fields.

The proposed framework is unvalidated. A prospective evaluation should record failed as well as successful identifications, compare predictions with the exposed nerve, and assess facial function at defined intervals.

## Figures and Tables

**Table 1:** Functional comparison of principal landmarks for facial nerve identification during parotidectomy.

Landmark	Anatomical Contribution	Typical Accessibility ^1^	Structural Behaviour ^2^	Operative Role and Principal Limitation	Evidence and Supporting References ^3^
Tympanomastoid suture	Deep osseous reference near the proximal trunk; reported distances vary	Directed exposure or palpation	Relatively fixed bone; the nerve may nevertheless be displaced	Refines deep anatomical orientation; proximity does not establish a safe dissection plane	C, I: [7,8,11,38]
Posterior belly of digastric muscle	Inferior reference toward the stylomastoid region	Often identifiable during regional exposure	Muscle position and contour may change with retraction or pathology	Provides inferior orientation; measurements depend on the selected border or attachment	C, I: [8,10,27,38]
Tragal pointer	Superficial cartilaginous directional reference	Early exposure	Mobile cartilage susceptible to deformation and retraction	Provides initial regional orientation; variable morphology and measurement origins limit precision	C, I: [9,11,36,38]
Mastoid tip	Posteroinferior osseous reference	Palpable during regional exposure	Relatively fixed bone	Provides broad orientation; separation from the trunk limits isolated localisation	C: [33]
Stylomastoid foramen	Direct exit of the facial nerve from the temporal bone	Deep; not routinely exposed initially	Fixed osseous aperture	Defines the proximal anatomical origin; direct exposure requires additional dissection	C: [40]
Styloid process	Deep reference within the stylomastoid region	Directed deep palpation or exposure	Relatively fixed bone; morphology varies	Provides supplementary deep orientation; restricted access limits routine use	C: [36]
Bony external auditory canal	Fixed regional reference adjacent to temporal-bone landmarks	Directed palpation or exposure	Rigid osseous component	Provides regional orientation; junctional measurements must not be attributed to the entire bony canal	A; C for junctional morphometry: [9]
Cartilaginous external auditory canal	Superficial regional reference, including the tragal cartilage	Early exposure	Mobile and deformable	Provides initial orientation; structural stability differs from that of the bony canal	A; C for junctional morphometry: [9]
Retromandibular vein	Indirect reference for the expected facial nerve plane	Preoperative imaging or deeper operative exposure	Compressible vessel with variable nerve relationships	Supports imaging and deep-plane orientation; does not define an invariant nerve plane	C: [56]; R, I: [73]
Posterior auricular, stylomastoid and occipital arteries	Vascular references around the proximal nerve and stylomastoid region	Directed identification	Vessel course and nerve relationships vary	Provide supplementary orientation; variation, small calibre and bleeding may complicate interpretation	C: [62,64]
Posterior auricular nerve	Direct branch of the proximal facial nerve	Targeted identification; may be obscured	Neural continuity does not imply resistance to scarring	Potential proximal tracing route; proposed usefulness in revision surgery lacks comparative clinical validation	C: [68,69]
Digastric branch of the facial nerve	Direct connection between the trunk and posterior belly of digastric	Targeted muscular and neural dissection	Small branch whose accessibility may be affected by fibrosis	Potential supplementary tracing route; clinical validation remains limited	C: [16]

^1^ Accessibility is an author synthesis of the stage and extent of exposure required. Operative exposure frequencies are given in Section 7 [38]. A landmark that could not be exposed contributes no distance measurement. ^2^ Structural behaviour: Assessments describe the expected mobility or deformation of the landmark itself. They do not establish preservation of its relationship to a displaced nerve or the presence of a safe dissection plane. These judgments incorporate anatomical interpretation and have not been measured uniformly in malignant, revision, irradiated or inflammatory settings. ^3^ Evidence: C, cadaveric; I, intraoperative; R, radiological; A, anatomical interpretation. References support the relevant anatomy, measurements or technique; they do not necessarily directly assess accessibility or resistance to distortion. For the external auditory canal, the study measured the bony–cartilaginous junction, rather than independently evaluating both canal components [9].

## Data Availability

The extracted study-level information and documented search strategies are available in the Supplementary Materials.

## Supplementary Materials

The following supporting information can be downloaded at: https://www.mdpi.com/article/10.3390/jcm15218718/s1. Table S1: Study-level morphometry; Table S2: Comparative surgery, monitoring and imaging; Supplementary Methods S1: Search history and record flow; Supplementary Register S1: Selected citation-to-claim checks. References [1,2,3,4,5,7,8,9,10,11,12,13,16,22,27,29,30,31,32,33,34,35,36,37,38,40,41,42,44,54,55,56,57,58,59,60,61,62,63,64,67,68,69,70,71,72,73,74,75,76,77,78,79,80] are cited in the Supplementary Materials and included in the main reference list.
