# Supplementary material for: Anatomical and Surgical Landmarks for Facial Nerve Identification During Parotidectomy: A Critical Narrative Review of Reliability, Clinical Utility, and Operative Decision-Making
Source: J Clin Med. 2026 Nov 8;15(21):8718. doi: 10.3390/jcm15218718 (PMC13764338; doi:10.3390/jcm15218718)
Supplement: Supplementary file 1 [file jcm-15-08718-s001.zip › jcm-4647279-supplementary.pdf]

Supplementary material

Table S1. Study-level morphometry

Twenty-two principal publications contribute 26 rows; four mixed studies have separate cadaveric and intraoperative rows. Donors, sides, patients and repeat observer measurements are different units. Unless indicated otherwise, linear measurements are in millimetres. SD, standard deviation; NV, not verified from the source material obtained. Source access refers to the verification undertaken during this revision: FT, full-text Methods/Results or tables consulted; A, abstract available but full text not obtained; P, partial source text available. NR means not reported in the accessible full text; unclear means the source notation could not be interpreted confidently. Neither is interchangeable with lack of access. Unlabelled ± values are retained without assuming that they represent SD.

Study, design and source access	Donors, sides or patients	Preparation	Landmark, neural endpoint and measurement	Results and interpretive limits
De Ru et al., 2001 — cadaveric morphometry [7]; access: FT	30 hemifaces; independent donor count NR. Endpoint-specific n=28–30; TMS n=29 per observer.	Formaldehyde-fixed	Tragal cartilage pointer (TP), PBDM and TMS. Trunk extends from SMF to first bifurcation. Shortest landmark-to-trunk distance, measured by two observers.	Mean ± SD, observers 1/2: TP 8.4 ± 3.6 (n=30)/7.3 ± 2.4 (n=28); PBDM 4.8 ± 2.27/4.5 ± 2.34 (n=29 each); TMS 2.7 ± 0.7/2.6 ± 0.8 (n=29 each). SD confirmed in Table I. Nerve exposed before measurement; observer correlation is not independent replication.
Witt, Weinstein and Rejto, 2005 — cadaveric [8]; access: A	14 cadaver specimens; independent donor count NV.	NV in accessible abstract	TMS and posterior-superior PBDM margin; closest measured distance to FN.	TMS mean 1.8 (range 0–4); PBDM mean 12.4 (7–17). Independent population from the clinical row. Preparation and exact point on TMS need full-text verification.
Witt, Weinstein and Rejto, 2005 — intraoperative [8]; access: A	22 patients.	Living tissue	TMS and posterior-superior PBDM margin; closest measured distance to FN.	TMS mean 2.0 (range 0–4); PBDM mean 10.7 (5–14). Does not isolate an effect of landmark selection on postoperative nerve function.
Pather and Osman, 2006 — cadaveric microdissection [36]; access: A	40 adult cadavers; measured side count NV in accessible abstract.	NV in accessible abstract	TP, PBDM attachment, TMS, EAC, styloid, transverse process of axis and mandibular angle. Shortest distances; precise points incompletely described in abstract.	Means (ranges): TP 34.0 (24.3–49.2); PBDM 14.6 (9.7–24.3); TMS 10.0 (4.9–18.6); EAC 13.4 (7.3–21.9); styloid 9.8 (4.3–18.6); axis 16.9 (9.7–36.8); mandibular angle 38.1 (25.3–48.69). Between-study disagreement cannot be assigned to a mechanism from abstract alone. Axis = C2, not C1.
Rea, McGarry and Shaw-Dunn, 2010 — comparative morphometry [9]; access: P	26 adult cadavers (15 female, 11 male); endpoint-specific hemiface n NV.	Embalmed, formaldehyde-fixed; confirmed in publisher Methods excerpt; full text not obtained	PBDM, TP, bony–cartilaginous junction of EAC, TMS; distances to main trunk. Finer point and trajectory definitions NV.	PBDM 5.5 ± 2.1; TP 6.9 ± 1.8; EAC junction 10.9 ± 1.7; TMS 2.5 ± 0.4. Mean age 83 years (59–98). Exact points and the ± dispersion label need full-text confirmation.
Saha et al., 2014 — cadaveric [10]; access: FT	10 fresh cadaveric dissections; independent donor count unspecified.	Fresh	TMS bone, TP and mastoid attachment of PBDM; landmark–trunk distances with slide callipers.	Reported means (ranges): TMS 3.5 (2.5–4.5); TP 16.61 (14–21); PBDM 7.41 in abstract versus 7.5 in Results (6–9.5). Source individual values are not fully concordant with summary TMS and PBDM estimates (note a).
Saha et al., 2014 — intraoperative [10]; access: FT	10 patients.	Living tissue	Same three landmarks; PBDM at mastoid attachment; landmark-to-trunk distance during parotidectomy.	Reported means (ranges): TMS 3.87 (2–6); TP 16.36 (13.6–19); PBDM 8.03 (6–11.5). Small sample. TP minimum is 13.5 in source Table 3 but 13.6 in prose.

Study, design and source access	Donors, sides or patients	Preparation	Landmark, neural endpoint and measurement	Results and interpretive limits
Stankevicius and Suchomlinov, 2019 — cadaveric [33]; access: FT	11 donors; 22 hemifaces; 20 in morphometry after two double trunks excluded.	10% formaldehyde solution	TP, mastoid tip, mandibular angle; shortest distance to trunk. Five digital-calliper measurements per point by one researcher.	Mean ± SD (range): TP 9.30 ± 0.93 (7.67–10.78); mastoid 12.53 ± 2.30 (8.99–17.26); angle 36.45 ± 4.14 (25.84–44.39). SD confirmed in Table 3. Mastoid abstract value is 12.52. Repeated readings and bilateral sides are not independent donors.
Kaushal et al., 2021 — intraoperative [11]; access: FT	60 patients: 54 superficial, 6 total conservative parotidectomies.	Living tissue	TP and palpated TMS without periosteal elevation; shortest distance to exposed main trunk using Castroviejo callipers.	TP 18.38 ± 6.85; TMS 2.92 ± 0.60. Recurrent disease and preoperative weakness excluded. TMS SD is identified in Discussion; TP dispersion is not explicitly defined. No comparative functional benefit established.
Shah et al., 2021 — cadaveric C–M–S [27]; access: P	41 cadavers; 82 parotid regions.	Preservative NR in accessible Methods	Fully exposed TP, superior PBDM border, palpated TMS; shortest distance to trunk with slide calliper.	TP 13.04 ± 5.238; TMS mean 6.50; PBDM mean 6.92. Meaning of the TP ± value and other parenthetical intervals remains unclear in accessible source text. Bilateral sides are dependent.
Shah et al., 2021 — intraoperative C–M–S [27]; access: P	20 patients.	Living tissue	Same cartilage–muscle–suture sequence; shortest distance to trunk with slide calliper.	TP 9.95 ± 3.967; TMS mean 6.35; PBDM mean 8.50. Dispersion notation remains unclear in accessible source text. Combined identification does not isolate a landmark effect or demonstrate reduced injury.
Verma et al., 2022 — cadaveric [35]; access: FT	14 hemifaces from 10 heads in Methods/Results; abstract states 12.	Fresh-frozen	Composite line between TMS and mastoid tip; line segment and its relation to FN crossing, not necessarily shortest isolated suture distance.	TMS–FN: prose mean 5 (2–7) vs Table 1 mean 10.42857. Mastoid–FN: Table 1 mean 8.5. TMS–mastoid: prose 17.24/17.26 vs Table 1 mean 18.92. Conflicting values unreconciled. Trunk divided before line in two specimens (note b).
Verma et al., 2022 — intraoperative [35]; access: FT	5 patients.	Living tissue	TMS-to-mastoid-tip line; distances among TMS, mastoid and FN trunk.	Means (ranges): TMS–FN 7.2 (4–10); mastoid–FN 11 (8–15); TMS–mastoid 18.6 (15–22). Identification in 4/5 patients = 80%; source inaccurately states 90%. Separate from cadaveric estimates.
Singh et al., 2025 — clinical [34]; access: FT	24 superficial-parotidectomy patients.	Living tissue	PBDM attachment (called insertion) and TP to exposed FNT, using vernier callipers. FN sought superior and medial to PBDM attachment.	Reported mean ± SD (range): PBDM 17 ± 0.87 (15–20); TP 23.5 ± 11.96 (5–43). SD explicitly identified in Results. Attachment differs from a closest superior-border point. Selected primary cohort; no causal nerve-preservation comparison.
Mendoza et al., 2014 — clinical [37]; access: FT	22 patients without preoperative paralysis; 21 superficial, 1 total.	Living tissue; supine, head turned contralaterally	Shortest distances from inferior/anterior TP and anterior palpable V-shaped TMS sulcus to extracranial FN entering parotid tissue; surgical legged calliper after nerve exposure, before mass excision.	Mean ± SD (range): TP 9.0 ± 2.8 (5–18); TMS 6.1 ± 2.0 (4–10). SD confirmed in Methods/Table 1. Single centre; measurement after nerve exposure. Head positioning and retraction may alter geometry.

Study, design and source access	Donors, sides or patients	Preparation	Landmark, neural endpoint and measurement	Results and interpretive limits
Sardar et al., 2021 — operative [38]; access: FT	32 patients with benign superficial-lobe masses; TP exposed in 32, PBDM in 26, TMS in 25.	Intraoperative	Exposed landmarks-to-trunk distances using spring callipers and graduated scale; preoperative ultrasound lesion volume.	Mean ± SD: TP 12.79 ± 2.33; PBDM 9.78 ± 1.21; TMS 7.58 ± 1.33. Entry required at least two landmarks. Tumour-volume correlations small and nonsignificant.
Salame et al., 2002 — cadaveric [40]; access: A	23 donors; bilateral dissection.	Formalin-fixed	Trunk diameter at SMF; length from SMF to first bifurcation.	Diameter 2.66 ± 0.55; length 16.44 ± 3.2. Nerve dimensions, not independent landmark separation or surgical safety margin. ± dispersion type NV.
Hewapathirana et al., 2006 — cadaveric [41]; access: A	28 head halves; independent donor n NV.	NV in accessible abstract	Tragal point, mastoid tip, caudal EAM, postglenoid tubercle, SMF and bifurcation. Several measures terminate at bifurcation rather than nearest trunk.	Tragal point–trunk 10.08 ± 2.34; –bifurcation 13.97 ± 2.72; bifurcation–mastoid 16.28 ± 2.87; –caudal EAM 19.64 ± 2.98; –postglenoid 23.83 ± 3.28; trunk length 18.51 ± 3.80; diameter at SMF 1.78 ± 0.31. Distinct endpoints not pooled; ± type NV.
Martínez Pascual et al., 2019 — cadaveric [42]; access: FT	23 donors; 38 dissectable facial nerves.	Embalmed; fixative NR	SMF emergence and bifurcation; trunk length and diameter.	Length 14.38 ± 3.04 (7.8–18.47); diameter 2.16 ± 0.49 (0.76–2.87). Trunk dispersion not explicitly labelled in Results, although SD is defined for distal-branch measurements; retained as unclear. Not landmark separation; 38 sides ≠ 38 donors.
Zhong and Ashwell, 2016 — branch morphometry [57]; access: FT	41 half-head specimens overall; RMV subsample 23 male and 7 female sides.	Prosected; preservation method NR	Highest RMV point below parotid to marginal mandibular branch after gland exit. Head extension 120°, mouth closed; three digital-calliper readings averaged.	Mean ± SD (range): male 10.2 ± 5.6 (1.9–19.3), n=23; female 12.4 ± 7.1 (1.5–24.8), n=7. SD confirmed in Table 3. Peripheral branch, not trunk; RMV subgroup differs from total specimen count.
Reddy et al., 2021 — arterial anatomy [64]; access: A	10 cadavers; 14 hemifaces.	NV in accessible abstract	PAA/stylomastoid branch relative to main FN trunk; precise arterial measurement origin needs full-text confirmation.	Mean PAA–FN separation 7 (range 2–14), as described in abstract (not median). Potential overlap with Verma cohort unresolved (note c).
Wen et al., 2026 — vascular anatomy [62]; access: FT	10 donors; 20 sides.	Freshly preserved; red silicone arterial perfusion	PAA–FN crossing, SMF, mastoid tip and OA–FN; specified paired points measured with calibrated vernier calliper (0.01 mm resolution).	Mean ± SD: PAA–mastoid 11.40 ± 2.16; origin–crossing 17.22 ± 2.44; crossing–SMF 14.95 ± 3.07; OA–FN 4.99 ± 1.09. SD confirmed in Methods/Table 1. Endpoints differ; perfusion may affect geometry; no clinical outcome comparison.
Kriengkraikasem et al., 2018 — cadaveric [67]; access: FT	16 donors; 31 sides (16 left, 15 right).	Preservation method NR	Widest GAN before anterior/posterior division and main FN trunk before bifurcation. Single observer; digital calliper (0.01 mm resolution). Width, not diameter.	Mean ± SD: GAN 3.26 ± 0.67; FN 3.36 ± 0.71; right r=0.740, left r=0.839. SD confirmed in Methods. Width correlation does not locate the nerve trajectory or demonstrate improved preservation.
Smith and Ross, 2012 — cadaveric [68]; access: A	6 donors; 11 facial sides.	Formalin-fixed	PAN origin from posterolateral FN trunk; distance from SMF to branch origin.	5.4 ± 3.3 (range 1.6–11.1). Course variable. Candidate superficial tracing route, without prospective revision validation. ± type NV.
Blau, Vaisbuch and Marom, 2019 — cadaveric [69]; access: A	75 heminecks; independent donor n NV.	NV in accessible abstract	PAN course from auricularis posterior muscle to FN trunk; length along nerve and angle to vertical FN segment.	Course length 28 ± 6.2; angle 39.5 ± 7.7°. Not straight-line separation; revision utility is proposed, not validated. ± type NV.

Study, design and source access	Donors, sides or patients	Preparation	Landmark, neural endpoint and measurement	Results and interpretive limits
Tayebi Meybodi et al., 2019 — cadaveric [16]; access: A	8 heads; 15 sides.	NV in accessible abstract	DBFN origin on extracranial trunk; insertion on superomedial PBDM; mastoid tip to DBFN entry into PBDM.	Mean ± SD (explicit in abstract): DBFN length 15.4 ± 3.4; mastoid–entry 13.6 ± 2.0 (10–17). Nerve entry point differs from muscle border-to-trunk distance. Clinical validation limited.

Notes to Table S1

a. Saha et al. report cadaveric PBDM mean 7.41 in the abstract and 7.5 in Results. Individual tabulated observations do not fully correspond to some reported TMS and PBDM summaries; no replacement estimates were calculated.

b. Verma et al. report conflicting cadaver sample counts and geometry between abstract, text and Table 1. Both numerical versions are retained when contradictory; this composite-line measure is not an isolated shortest TMS-to-trunk distance.

c. Reddy et al. and Verma et al. share investigators and the reported cadaver/hemiface counts. Dataset independence cannot be established, so they should not automatically be counted as distinct anatomical cohorts.

Abbreviations: DBFN, digastric branch of facial nerve; EAC/EAM, external auditory canal/meatus; FN, facial nerve; GAN, great auricular nerve; OA, occipital artery; PAA, posterior auricular artery; PAN, posterior auricular nerve; PBDM, posterior belly of digastric muscle; RMV, retromandibular vein; SMF, stylomastoid foramen; TMS, tympanomastoid suture; TP, tragal pointer.

Table S2. Comparative surgery, monitoring and imaging

Direction of comparison and endpoint definitions are specified in each row. OR, odds ratio; RR, risk ratio; CI, confidence interval; H–B, House–Brackmann; EMG, electromyography; MR, magnetic resonance. Estimates were not pooled in this review, and the meta-analyses overlap in primary reports.

Report	Population and comparison	Endpoint	Results	Limits or reference standard
Stankovic et al., 2018 [29]	8 publications; arm denominators not in abstract.	Anterograde vs retrograde; H–B palsy.	Temporary OR 2.64 (95% CI 0.97–7.21); permanent OR 4.31 (0.44–42.28).	Wide CIs; no equivalence test or validated scarred-field benefit.
Mashrah et al., 2018 [30]	10 reports, 570 patients: 319 retrograde, 251 antegrade; palsy analysis n=481.	Benign surgery; antegrade vs retrograde.	Transient RR 1.16 (0.80–1.67); permanent RR 4.63 (0.72–29.04).	Excludes revision, recurrence and malignancy; overlapping meta-analyses.
Smith et al., 2025 [31]	35 retrograde and 70 anterograde patients; benign superficial parotidectomy.	Temporary paresis.	3/35 vs 30/70; p<0.001.	Retrospective, non-randomised; size and site balance cannot remove selection or histological confounding.
Kim, Jang and Hwang, 2026 [32]	21 studies; 1,632 patients.	Retrograde vs antegrade.	Transient palsy OR 0.73 (0.54–0.98), principally superficial surgery.	No significant permanent-palsy difference; benign surgery, not revision-field evidence.
Sharma et al., 2011 [72]	39 patients: 29 proximal and 10 distal explorations during superficial parotidectomy.	Distal buccal exploration because fibrosis hindered proximal trunk identification.	Feasible in selected difficult fields; no adjusted comparative estimate for nerve safety.	Retrospective; routes selected by operative difficulty, not proof of revision/irradiated-field superiority.
Grosheva et al., 2009 [77]	100 primary operations in 96 patients; 50 monitored, 50 control.	EMG plus visual assessment vs visual only.	No significant immediate or definitive facial-function difference (p=0.23; p=0.45).	Primary benign/inflammatory surgery; monitoring adjunct does not substitute for anatomical identification.
Kucharska et al., 2024 [71]	72 revision patients; 109 operations, mixed benign and malignant.	H–B status before and ≥2 years after last revision.	Table II: 40/72 grade I (55.6%); 32/72 grade II–VI (44.4%).	Prose and Table II disagree; claimed deterioration 27/73 conflicts with 72-patient sample. Final status ≠ incident palsy.
Psychogios et al., 2019 [73]	50 patients with benign tumours.	Binary tumour–nerve contact; predicted versus performed surgery.	Contact 48/50 (96%); planned/performed surgical-technique agreement 49/50 (98%).	Reference: operative findings/photos and operation performed; surgeon blinded to predictions; no numeric contact threshold or demonstrated preservation effect.
Jeong et al., 2023 [74]	296 participants; 120 evaluable scans; outcomes 105 MRI vs 165 non-MRI; matched 65 per group.	Direct MR depiction, surgical localisation and function.	Trunk visible 117/120 (97.5%); tumour site agreed 109/120 (90.8%). Matched median identification 25 vs 35 min. Temporary palsy OR 2.29 (95% CI 0.64–8.25); permanent OR 2.02 (0.32–12.90), MRI vs non-MRI.	Intentional nerve sacrifice excluded from outcomes. Non-randomised and propensity-adjusted; wide CIs cannot establish equivalence.
Gaudino et al., 2024 [75]	40 patients; independent imaging readers and surgical correlation.	MR localisation.	Distal nerve visibility 34%; surgical agreement trunk/temporofacial/cervicofacial 100%/96%/89%.	Agreement ≠ controlled nerve preservation.
Hu et al., 2024 [78]	15 patients.	Segmentation, mixed reality and navigation feasibility.	Trunk visualised in 15/15; investigators classified 36/37 sampled points correctly segmented.	Point-level classification; acceptance tolerance and independent adjudication NV; no controlled functional comparison.

Supplementary Methods S1. Search history and record flow

The original review covered database inception through 7 October 2026. The historical database histories and stage-specific screening counts were not retained. Non-English reports were eligible when assessable in translation. A PubMed retrieval check on 18 October and further three-database exports reconciled on 2 November are documented below. Two authors confirm that they screened all 7,157 deduplicated November records by title and abstract, assessed relevant full texts and jointly decided eligibility. No additional studies were incorporated from this screening. One contextual zygomaticus major imaging report [22] was added during revision; the morphometric evidence set remains unchanged. The workbook eligibility fields remained blank, so this account of screening cannot be reconstructed from individual recorded decisions. Full-text assessment and exclusion totals are unavailable; no retrospective systematic-review flow is claimed.

Activity	Source records	What the count represents
Historical narrative selection	Historical retrieval and exclusion counts unavailable.	Two authors screened titles, abstracts and relevant full texts, extracted evidence and made joint eligibility decisions.
18 Oct 2026 — PubMed retrieval check	3,312 records, publication dates through 7 Oct.	Known-paper retrieval validation. All 19 DOI-matched PubMed records from the then 20-report morphometry table were retrieved. Two later Table S1 additions were not part of that check.
2 Nov 2026 — three-database exports	PubMed 3,991; Scopus 6,538; Web of Science Core Collection 3,012; total 13,541.	Reconciliation date. Seven Web of Science export files bear 2 Nov. Raw exports do not establish exact execution times or interface-only filters for other sources.
Deduplication	6,384 duplicate rows; 7,157 distinct records.	Matched PMID/normalised DOI and bibliographic features; ambiguous pairs manually checked. Abstracts were present for 5,999 retained records and absent for 1,158. Screening and relevant full-text assessment are author-confirmed; individual decisions were not recorded in the workbook.
Screening and synthesis	7,157 November records screened; no additional studies incorporated from screening. Full-text assessment and exclusion totals unavailable.	Two authors confirm title/abstract screening, assessment of relevant full texts and joint eligibility decisions. November eligibility fields were not populated. The 22 morphometric publications, 26 table rows and 80 distinct cited publications describe the synthesis, not reconstructed screening-stage counts. The contextual imaging citation added during revision is separate from the November screening yield.

S1.1. PubMed — executed retrieval check, 18 October 2026

NCBI ESearch. Publication date through 7 October 2026. No language, publication-type or species filter. A technical lower year of 1000 imposes no relevant publication-date restriction.

("Facial Nerve"[MeSH Terms] OR "facial nerve"[Title/Abstract] OR "facial nerves"[Title/Abstract]) AND ((("Parotid Gland"[MeSH Terms] OR "Parotid Neoplasms"[MeSH Terms] OR parotid*[Title/Abstract]) AND (landmark*[Title/Abstract] OR anatom*[Title/Abstract] OR morphometr*[Title/Abstract] OR identif*[Title/Abstract] OR localiz*[Title/Abstract] OR localis*[Title/Abstract] OR dissect*[Title/Abstract] OR retrograd*[Title/Abstract] OR antegrad*[Title/Abstract] OR anterograd*[Title/Abstract] OR monitor*[Title/Abstract] OR stimulat*[Title/Abstract] OR imag*[Title/Abstract] OR ultrason*[Title/Abstract] OR sonograph*[Title/Abstract] OR neurograph*[Title/Abstract] OR navigat*[Title/Abstract] OR fluorescen*[Title/Abstract] OR preserv*[Title/Abstract] OR injur*[Title/Abstract] OR palsy[Title/Abstract] OR paresis[Title/Abstract] OR paralysis[Title/Abstract] OR dysfunction[Title/Abstract] OR outcome*[Title/Abstract])) OR ("tragal pointer"[Title/Abstract] OR tympanomastoid[Title/Abstract] OR "posterior belly of digastric"[Title/Abstract] OR "digastric muscle"[Title/Abstract] OR "mastoid tip"[Title/Abstract] OR "mastoid process"[Title/Abstract] OR "stylomastoid foramen"[Title/Abstract] OR "styloid process"[Title/Abstract] OR "retromandibular vein"[Title/Abstract] OR "posterior auricular artery"[Title/Abstract] OR "posterior auricular nerve"[Title/Abstract] OR "great auricular nerve"[Title/Abstract] OR "greater auricular nerve"[Title/Abstract] OR "digastric branch"[Title/Abstract])) AND ("1000/01/01"[Date - Publication] : "2026/10/07"[Date - Publication])

S1.2. November three-database search and screening — documented strategies

The accompanying November search protocol contains the strings below. No date or language restriction is encoded. Exact pasted search histories and any additional interface selections cannot be inferred from exported files. These strategies do not reconstruct the original October search.

PubMed — MeSH and title/abstract

("Facial Nerve"[Mesh] OR "facial nerve*"[tiab] OR "seventh cranial nerve"[tiab] OR "cranial nerve VII"[tiab] OR "marginal mandibular"[tiab] OR "buccal branch*"[tiab] OR "zygomatic branch*"[tiab] OR "temporal branch*"[tiab] OR "cervical branch*"[tiab]) AND ("Parotid Gland"[Mesh] OR "Parotid Neoplasms"[Mesh] OR parotid*[tiab] OR intraparotid*[tiab] OR "tragal pointer*"[tiab] OR tympanomastoid*[tiab] OR "tympano-mastoid"[tiab] OR stylomastoid*[tiab] OR retromandibular[tiab] OR "posterior auricular arter*"[tiab] OR (posterior[tiab] AND digastric[tiab]) OR ((extratemporal[tiab] OR extracranial[tiab] OR "facial nerve trunk*"[tiab]) AND (landmark*[tiab] OR morphometr*[tiab] OR topograph*[tiab] OR "mastoid tip"[tiab] OR "styloid process"[tiab] OR "external auditory canal"[tiab] OR "external auditory meatus"[tiab] OR "external acoustic meatus"[tiab])))

Scopus — title, abstract and keywords

TITLE-ABS-KEY(("facial nerve*" OR "seventh cranial nerve" OR "cranial nerve VII" OR "marginal mandibular" OR "buccal branch*" OR "zygomatic branch*" OR "temporal branch*" OR "cervical branch*") AND (parotid* OR intraparotid* OR "tragal pointer*" OR tympanomastoid* OR "tympano-mastoid" OR stylomastoid* OR retromandibular OR "posterior auricular arter*" OR (posterior AND digastric) OR ((extratemporal OR extracranial OR "facial nerve trunk*") AND (landmark* OR morphometr* OR topograph* OR "mastoid tip" OR "styloid process" OR "external auditory canal" OR "external auditory meatus" OR "external acoustic meatus"))))

Web of Science Core Collection — Topic

TS=(("facial nerve*" OR "seventh cranial nerve" OR "cranial nerve VII" OR "marginal mandibular" OR "buccal branch*" OR "zygomatic branch*" OR "temporal branch*" OR "cervical branch*") AND (parotid* OR intraparotid* OR "tragal pointer*" OR tympanomastoid* OR "tympano-mastoid" OR stylomastoid* OR retromandibular OR "posterior auricular arter*" OR (posterior AND digastric) OR ((extratemporal OR extracranial OR "facial nerve trunk*") AND (landmark* OR morphometr* OR topograph* OR "mastoid tip" OR "styloid process" OR "external auditory canal" OR "external auditory meatus" OR "external acoustic meatus"))))

Reconciliation. Records were reconciled by PMID and normalised DOI, followed by normalised title plus author/year or journal volume and initial page. Ambiguous candidates were checked individually. Notices, separate publications and potentially overlapping cohorts were kept distinct when indicated. The source 13,541 rows were linked to 7,157 deduplicated records in the search workbook.

Supplementary Register S1. Selected citation-to-claim checks

Reference numbers follow the corrected manuscript bibliography [1–80]. This register records selected citation-to-claim checks and is not a replacement bibliography. DOI links (or PubMed links when no DOI is supplied) identify the source. Tables S1 and S2 distinguish verified details from remaining methodological uncertainty.

Reference	Claim supported / endpoint or limit	Source
[1] Alomar OSK	Observed variation in trunk division and branching during parotidectomy.	DOI
[2] Davis RA	Anatomical study of 350 cervicofacial halves; halves are not necessarily independent donors.	PubMed
[3] Katz AD	Intraparotid nerve anastomoses in an operative series.	DOI
[4] Ruohoalho J	Prospective benign cohort; facial function measured at multiple postoperative intervals.	DOI
[5] Gaillard C	Primary conservative procedures; extent of surgery and immediate versus later weakness.	DOI
[7] De Ru JA	Two observers, one anatomical sample; independent readings are not extra specimens.	DOI
[8] Witt RL	Suture and posterior-superior muscle-margin distances in separate cadaveric and clinical samples.	DOI
[9] Rea PM	Four landmark relationships in fixed cadavers; 10.9 mm refers to EAC bony–cartilaginous junction.	DOI

Reference	Claim supported / endpoint or limit	Source
[10] Saha S	Fresh cadaveric and intraoperative measurements; discrepancies retained explicitly.	DOI
[11] Kaushal D	Intraoperative TMS and TP distances; measurement precision does not establish better outcomes.	DOI
[12] Ji YD	Earlier systematic review of landmark-to-trunk distances, used as synthesis comparator.	DOI
[13] Łabętowicz P	Broader narrative review of surgical FN anatomy, distortion and preservation.	DOI
[16] Tayebi Meybodi A	Digastric-branch tracing; branch length/muscle entry differ from trunk separation.	DOI
[27] Shah AS	Published cartilage–muscle–suture sequence; feasibility not evidence of functional benefit.	DOI
[29] Stankovic P	Earlier antegrade-versus-retrograde meta-analysis with imprecise permanent outcome.	DOI
[30] Mashrah MA	Benign-surgery meta-analysis excluding revision/recurrent/malignant settings.	DOI
[31] Smith JD	Benign superficial operations; temporary palsy, comparison controlling for size and site.	DOI
[32] Kim DH	Updated benign-surgery meta-analysis; transient and permanent outcomes distinct.	DOI
[33] Stankevicius D	Mastoid-tip distance and exclusions; abstract/table difference of 0.01 mm.	DOI
[34] Singh A	PBDM attachment and TP distances in 24 selected primary patients.	DOI
[35] Verma R	Composite TMS–mastoid geometry; conflicting source sample sizes, measures and percentage.	DOI
[36] Pather N	TMS mean 10.0 mm (4.9–18.6), crucial for between-study reproducibility.	DOI
[37] Mendoza DJC	Intraoperative distances, explicitly reported SDs and observed ranges.	DOI
[38] Sardar S	Conditional distances and exposure frequency; eligibility required two landmarks.	DOI
[40] Salame K	Calibre and length of FN at SMF; not a landmark-to-trunk distance.	DOI
[41] Hewapathirana TN	Morphometry with differing anatomical endpoints and bifurcation measurements.	DOI
[42] Martínez Pascual P	Cadaveric FN course and dimension measurements.	DOI
[44] Joseph ST	Composite styloid base and digastric-origin localisation method.	DOI
[54] Ozgur Z	Atypical stylohyoid insertions in 22/56 sides; not tendon-penetration prevalence.	DOI
[55] Poutoglidis A	FN–RMV relation in 24 hemifaces.	DOI
[56] Touré G	FN–RMV configuration in 132 parotid glands, including nonbinary relationships.	DOI
[57] Zhong W	Highest subparotid RMV point to marginal mandibular branch; distal endpoint.	DOI
[58] Landfald IC — facial artery	Cervical facial-artery variation for vascular context; not nerve localisation.	DOI
[59] Landfald IC — lingual artery	Scoping review; common-trunk prevalence varies across contributing reports.	DOI
[60] El-Hakim H	Cross-sectional radiological prediction of lesion–nerve plane.	DOI
[61] Upile T	Stylomastoid-artery landmarks in clinical and anatomical material.	DOI
[62] Wen F	Proximal FN–PAA vascular variation in 20 cadaveric sides.	DOI
[63] Liu M	Posterior auricular artery anatomical relationship in cadaveric hemifaces.	DOI
[64] Reddy GGSS	PAA-to-FN distance; potential cohort overlap with Verma needs disclosure.	DOI
[67] Kriengkraikasem K	GAN and FN-trunk widths; width correlation does not locate trunk trajectory.	DOI
[68] Smith OJ	PAN origin distance from SMF.	DOI
[69] Blau I	Posterior auricular nerve tracing and measured course length.	DOI
[70] Cho WK	Parotid carcinoma: imaging prediction of pathologically established FN involvement.	DOI
[71] Kucharska E	Final H–B status, and conflicting deterioration denominator in revision series.	DOI
[72] Sharma R	Distal tracing selected after proximal fibrosis encountered; strong allocation bias.	DOI
[73] Psychogios G	Binary tumour–nerve contact and performed-surgery agreement are not functional outcomes.	DOI
[74] Jeong HS	MR neurography with operative localisation, time, and adjusted functional comparison.	DOI
[75] Gaudino C	MR neurography; distal visibility, operative correlation and function distinct.	DOI
[76] Scharpf J	Consensus recommendations and troubleshooting for electrophysiological monitoring.	DOI
[77] Grosheva M	Two-centre primary-surgery monitoring comparison, no significant facial-function effect.	DOI
[78] Hu LH	Navigation feasibility and point-level segmentation, tolerance not confirmed.	DOI
[79] Hussain T	Mouse-model fluorescent peptide and whisker function; preclinical evidence only.	DOI
[80] Chiesa-Estomba CM	Postoperative palsy prediction, distinct from direct nerve localisation.	DOI
